# Metformin Attenuates TGF-β1-Induced Fibrosis in Salivary Gland: A Preliminary Study

**DOI:** 10.3390/ijms242216260

**Published:** 2023-11-13

**Authors:** Lianhao Wang, Nian-Nian Zhong, Xiaofeng Wang, Boyuan Peng, Zhuo Chen, Lili Wei, Bo Li, Yuhong Li, Yong Cheng

**Affiliations:** 1 State Key Laboratory of Oral & Maxillofacial Reconstruction and Regeneration, Key Laboratory of Oral Biomedicine Ministry of Education, Hubei Key Laboratory of Stomatology, School & Hospital of Stomatology, Wuhan University, Wuhan 430079, China; 2 Department of Oral Radiology, School & Hospital of Stomatology, Wuhan University, Wuhan 430079, China

**Keywords:** submandibular gland, fibrogenesis, NADPH Oxidase 4, reactive oxygen species, AMP-activated protein kinases, fibroblast

## Abstract

Fibrosis commonly arises from salivary gland injuries induced by factors such as inflammation, ductal obstruction, radiation, aging, and autoimmunity, leading to glandular atrophy and functional impairment. However, effective treatments for these injuries remain elusive. Transforming growth factor-beta 1 (TGF-β1) is fundamental in fibrosis, advancing fibroblast differentiation into myofibroblasts and enhancing the extracellular matrix in the salivary gland. The involvement of the SMAD pathway and reactive oxygen species (ROS) in this context has been postulated. Metformin, a type 2 diabetes mellitus (T2DM) medication, has been noted for its potent anti-fibrotic effects. Through human samples, primary salivary gland fibroblasts, and a rat model, this study explored metformin’s anti-fibrotic properties. Elevated levels of TGF-β1 (*p* < 0.01) and alpha-smooth muscle actin (α-SMA) (*p* < 0.01) were observed in human sialadenitis samples. The analysis showed that metformin attenuates TGF-β1-induced fibrosis by inhibiting SMAD phosphorylation (*p* < 0.01) through adenosine 5′-monophosphate (AMP)-activated protein kinase (AMPK)-independent pathways and activating the AMPK pathway, consequently suppressing NADPH oxidase 4 (NOX4) (*p* < 0.01), a main ROS producer. Moreover, in rats, metformin not only reduced glandular fibrosis post-ductal ligation but also protected acinar cells from ligation-induced injuries, thereby normalizing the levels of aquaporin 5 (AQP5) (*p* < 0.05). Overall, this study underscores the potential of metformin as a promising therapeutic option for salivary gland fibrosis.

## 1. Introduction

Fibrosis, as a predominant pathological manifestation, is crucial in salivary gland diseases. It arises frequently in conditions such as chronic inflammation, ductal obstruction, aging, radiation, and autoimmune damage of the salivary gland [1]. Notably, fibrosis is characterized by the proliferation of salivary gland fibroblasts, excessive production, and accumulation of extracellular matrix (ECM). Simultaneously, acinar cells undergo atrophy and demise owing to various pathological stimuli [2]. Both progressive interstitial fibrosis and parenchymal cell atrophy are salient indicators of pathological remodeling, leading to diminished glandular function. Contemporary clinical treatments for salivary gland diseases—encompassing endoscopy, Botox injections, and surgical interventions—have not yielded consistently promising outcomes [3]. As such, understanding the molecular mechanisms behind fibrosis progression and identifying effective pharmacological inhibitors remain paramount in salivary gland disease therapy.

The transforming growth factor-beta 1 (TGF-β1) stands out as a central proponent of fibrosis [4]. It primarily operates via SMAD-dependent or independent pathways under the governance of an intricate network of co-receptors and interactions [5]. Particularly, the TGF-β1-SMAD2/3 signaling cascade has been implicated in the advancement of salivary gland fibrosis. For instance, murine models exhibiting submandibular duct ligation displayed significant upregulation of TGF-β1, its receptor, and downstream SMAD transcription factors within the submandibular glands (SMG) [6]. Simultaneously, the TGF-β1/SMAD/Snail signaling axis has been shown to modulate epithelial–mesenchymal transition (EMT)-dependent fibrosis in primary Sjogren’s syndrome (PSS) salivary gland epithelial cells (SGEC) [7]. Moreover, TGF-β1 and interleukin (IL)-4 IL-10 levels surged in cases of IgG4-related Sialadenitis [8]. Recent research indicates reactive oxygen species (ROS) as mediators for TGF-β1 via pathways like the SMAD, mitogen-activated protein kinase (MAPK), and HO-GTPase [9]. Our prior investigations highlighted a marked increase in oxidative damage indicators, such as 4-hydroxynonenal (HNE) and malondialdehyde (MDA), particularly in collagen-rich zones of chronic sialadenitis (CS) [10]. These findings intimate ROS’s possible involvement in glandular fibrosis, warranting more detailed exploration.

NADPH oxidases (NOXs) encompass heme-containing transmembrane proteins, recognized as vital ROS generators in both phagocytic and non-phagocytic cells. Among the NOX family, NOX4’s role in TGF-β1-induced fibrosis has been extensively researched. Accumulated evidence reveals TGF-β1-stimulated NOX4 expression across diverse cell types [11,12,13]. ROS generated by NOX4 bolster the fibrogenic functions of TGF-β1 on fibroblasts, such as their differentiation into myofibroblasts [14,15,16]. Nonetheless, NOX4’s role in the context of TGF-β1-driven salivary gland fibrosis remains under-explored.

Metformin, a widely used biguanide for type 2 diabetes mellitus (T2DM) management, has recently been lauded for its anti-fibrotic properties. Studies have illuminated metformin’s capacity to reverse pulmonary fibrosis, invoking myofibroblast inactivation and apoptosis through adenosine 5′-monophosphate (AMP)-activated protein kinase (AMPK) phosphorylation [17]. AMPK, when recognized as a cellular energy sensor, becomes activated under energy–stress conditions. Its activation facilitates ATP production pathways, thereby re-establishing cellular homeostasis. Notably, AMPK activators have been found to diminish the production of ROS, specifically those derived from NOX4 and NOX2 [18]. Concurrently, metformin exhibits the potential to attenuate peritoneal fibrosis through its modulation of oxidative stress [19]. Furthermore, clinical findings affirm that metformin administration results in a notable reduction in ovarian collagen tissue and fibrosis in postmenopausal patients diagnosed with T2DM [20]. In the context of radiation-induced salivary gland dysfunction, metformin demonstrates efficacy in curtailing compensatory proliferation and reinstating salivary flow rates to benchmarks observed in unirradiated controls [21]. Based on these insights, we posit metformin as a promising therapeutic candidate against salivary gland fibrosis.

In our present investigation, we employ human clinical specimens, primary salivary gland fibroblasts, and rat models simulating submandibular gland fibrosis. Our objective is to delineate the therapeutic role and underlying mechanism of metformin in mitigating fibrosis associated with chronic salivary gland inflammation.

## 2. Results

### 2.1. Evaluation of Fibrotic Response in Human Chronic Sialadenitis

**Figure 1:**
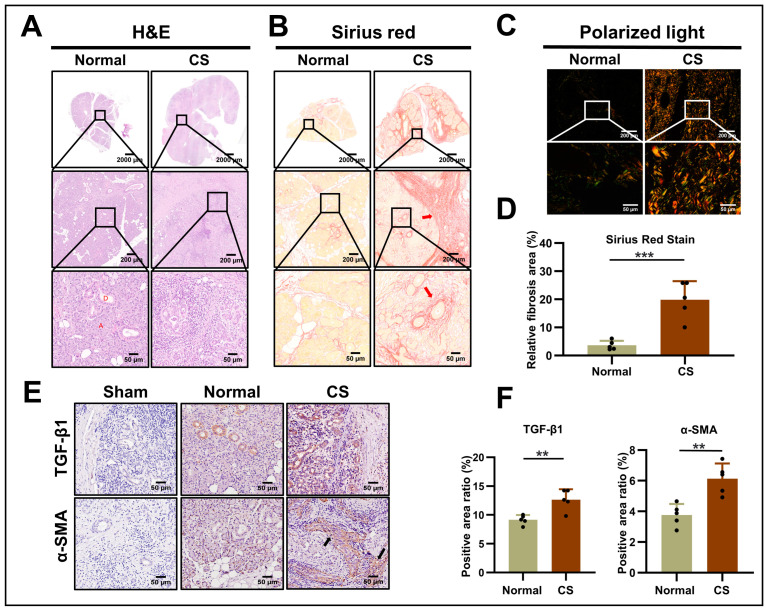
Evaluation of fibrotic response in human CS: (**A**) H&E staining of human SMGs. Middle panels display enlarged images (scale bar: 200 µm) obtained from the boxes in the upper panels (scale bar: 2000 µm), and lower panels show further magnified images (scale bar: 50 µm). (**B**) Sirius red staining of fibrotic SMGs. Arrows indicate the fibrosis surrounding the blood vessels and interlobular spaces. (**C**) Polarized light microscopy illustrating glandular fibrosis. Enlarged images (scale bar: 50 µm) are obtained from boxes in the upper panels (scale bar: 200 µm). (**D**) Quantification of the relative fibrotic area in normal (*n* = 5) and CS (*n* = 5) groups. Data are presented as means ± SD; *** *p* < 0.001. (**E**) Expression of TGF-β1 and α-SMA in human SMG (scale bar: 50 µm). Arrows indicate areas of positive expression. (**F**) Ratio of the positively stained area for TGF-β1 (*n* = 5) and α-SMA (*n* = 5). Data are represented as means ± SD; ** *p* < 0.01. H&E, hematoxylin and eosin; CS, chronic sialadenitis; A, acini; D, duct; TGF-β1, transforming growth factor-beta 1; α-SMA, alpha-smooth muscle actin; SMGs, submandibular glands; SD, standard deviation.

In normal human SMGs, hematoxylin and eosin (H&E) staining clearly delineated glandular lobules and ducts by fibrous connective tissue. Conversely, human CS tissues exhibited acinar cell atrophy, increased infiltration of inflammatory cells, ductal cell proliferation, and abundant collagen fiber deposition (Figure 1A). Sirus red staining indicated that the majority of collagen fibers were deposited in interlobular tissue, exhibiting a swirling pattern centered around ducts and blood vessels, while a smaller proportion was found in intralobular tissue, demonstrating a similar distribution (Figure 1B). Under polarized light, there was a notable enhancement of yellow and orange birefringence, while green birefringence was less intense. Predominantly, the deposited collagen fibers were type I collagen, accompanied by type III collagen (Figure 1C). Semi-quantitative analysis revealed a significantly greater quantity of fibers in CS tissue compared to normal tissue (*p* < 0.001) (Figure 1D). Concurrently, immunohistochemical (IHC) staining demonstrated a faint coloration of TGF-β1 in the ductal cells of normal SMG, whereas in CS glands, TGF-β1 was positively expressed not only in the ductal cells but also in the proliferative fibrotic tissue areas (*p* < 0.01). Similarly, α-SMA, a myofibroblast marker, was exclusively expressed in the peripheral area of acinar cells in normal SMG. However, in CS tissues, strong positive reactions of α-SMA were also observed in regions with collagen deposition (*p* < 0.01) (Figure 1E,F).

### 2.2. Characteristics of Human Primary Salivary Gland Fibroblasts (HPSFs)

**Figure 2:**
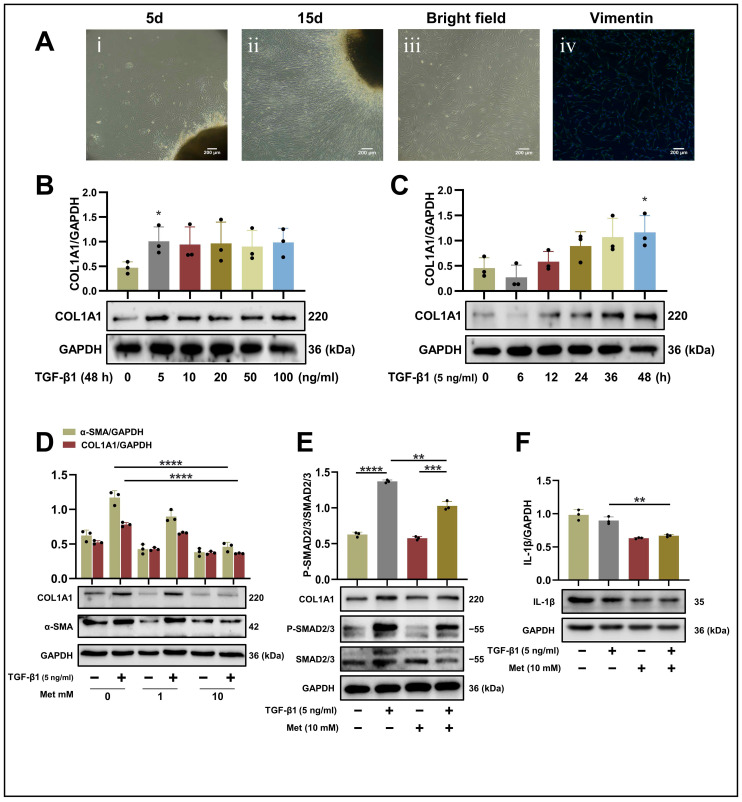
Metformin attenuates the fibrotic effects of TGF-β1 in HPSFs: (**A**) (**i**,**ii**) Cell growth surrounding the tissue explant observed at 5 and 15 days using an inverted microscope. (**A**) (**iii**) Isolated pure fibroblasts following multiple rounds of detachment and adherence. (**A**) (**iv**) Representative images depicting vimentin (green) and nuclei (blue) in fibroblasts (scale bar: 200 µm). (**B**,**C**) Fibroblasts were exposed to TGF-β1, and cell lysates were analyzed via SDS-PAGE and Western blot analysis for COL1A1 and GAPDH. (**B**) TGF-β1 induced a dose-dependent increase in COL1A1 production by salivary gland fibroblasts at 48 h (*n* = 3 per group). (**C**) TGF-β1 (5 ng/mL) stimulated a time-dependent increase in COL1A1 production by salivary glands fibroblasts (*n* = 3 per group). * *p* < 0.05 vs. control cells (0 ng/mL or 0 h). (**D**) Western blot analysis using anti-COL1A1, anti-α-SMA, anti-P-AMPK, and anti-GAPDH antibodies on cell lysates from control (lane 1, 2), 1 mM metformin (lane 3, 4), and 10 mM metformin (lane 5, 6) treated HPSFs. Metformin treatment commenced 24 h prior to TGF-β1 (5 ng/mL) stimulation, and protein samples were harvested following 48 h of TGF-β1 treatment. Upper panels display the average (±SD) relative expression from three independent experiments. ns > 0.05 and **** *p* < 0.0001. (**E**,**F**) Western blot analysis using anti-COL1A1, anti-SMAD2/3, anti-phospho-SMAD2/3 (P-SMAD2/3), anti-IL-1β and anti-GAPDH antibodies on cell lysates from control (lane 1, 2), and 10 mM metformin (lane 3, 4) treated HPSFs. Metformin treatment began 24 h before TGF-β1 (5 ng/mL) stimulation and protein samples were collected after 48 h of TGF-β1 treatment. Upper panels show the average (±SD) relative expression from three independent experiments. ** *p* < 0.01, *** *p* < 0.001, **** *p* < 0.0001. Met, metformin; TGF-β1, transforming growth factor-beta 1; COL1A1, collagen type I alpha 1; P-AMPK, phospho-adenosine 5′-monophosphate (AMP)-activated protein kinase; IL-1β, interleukin-1β; GAPDH, glyceraldehyde-3-phosphate dehydrogenase; HPSFs, human primary salivary gland fibroblasts; SDS-PAGE, sodium dodecyl sulfate–polyacrylamide gel electrophoresis.

Following tissue explant attachment, epithelial cells were initially observed in the culture dish, succeeded by the appearance of fibroblasts, as viewed under a CKX53 inverted microscope (Olympus). By the fifth day, both epithelial cells and fibroblasts were present concurrently (Figure 2A(i)). By the fifteenth day, the culture dish was completely populated with cells (Figure 2A(ii)). Utilizing the distinct detachment and adherence times of epithelial cells and fibroblasts, multiple rounds of adherence facilitated the isolation of relatively pure fibroblast populations. These isolated fibroblasts exhibited a characteristic spindle shape with numerous protrusions (Figure 2A(iii)). To ascertain the purity of the isolated fibroblasts, immunofluorescence (IF) staining was employed to assess vimentin expression in the primary cultured cells. Fluorescence microscopy images revealed that the fibroblast purity exceeded 95% (Figure 2A(iv)).

### 2.3. Effect of TGF-β1 on Collagen Type I Alpha 1 (COL1A1) Production in HPSFs

To investigate the ability of TGF-β1 to induce collagen production in HPSFs, cells were exposed to varying concentrations (0, 5, 10, 20, 50, 100 ng/mL) and durations (0, 6, 12, 24, 36, 48 h) of TGF-β1. Production of COL1A1 in the fibroblasts was quantified using Western blot analysis. The findings demonstrated that TGF-β1 stimulated COL1A1 production in HPSFs in a concentration-dependent manner. Specifically, at 48 h, the expression of COL1A1 induced by 5 ng/mL TGF-β1 exhibited a significant difference (*p* < 0.05), whereas the expression did not increase significantly with higher concentrations (Figure 2B). Moreover, at a concentration of 5 ng/mL, TGF-β1 triggered a time-dependent increase in COL1A1 production, reaching a peak at 48 h (*p* < 0.05) (Figure 2C). Consequently, a TGF-β1 concentration of 5 ng/mL and a duration of 48 h were selected for subsequent cell model analyses.

### 2.4. Metformin Reduces COL1A1 Production by Inhibiting the TGF-β1-SMAD2/3 Pathway and Interleukin Production in HPSFs

To assess whether metformin inhibits the activation of TGF-β1 in HPSFs and to identify the optimal concentration for its effect, cells were pretreated with 1 mM and 10 mM metformin for 24 h. Subsequently, the expression levels of fibrosis-related proteins were analyzed using Western blot technology. The findings revealed that 10 mM metformin significantly enhanced the phosphorylation of AMPK. Moreover, at this concentration, metformin notably suppressed the expression of COL1A1 (*p* < 0.0001) and α-SMA (*p* < 0.0001) (Figure 2D). The anti-fibrotic effects of metformin at different concentration gradients have been further demonstrated (Supplementary Figure S1A). TGF-β1 stimulation markedly elevated the levels of phosphorylated SMAD2/3 (*p* < 0.0001), an effect that was mitigated by metformin (*p* < 0.01) (Figure 2E). Based on the fact that IL-1β can potentiate the role of TGF-β in the fibrotic response [22], we also examined the expression of IL-1β during metformin therapy. The results demonstrated that 10 mM metformin significantly decreased IL-1β production in HPSFs (*p* < 0.01) (Figure 2F).

### 2.5. NOX4 Is Involved in the Fibrotic Process in HPSFs by Regulating ROS Levels

**Figure 3:**
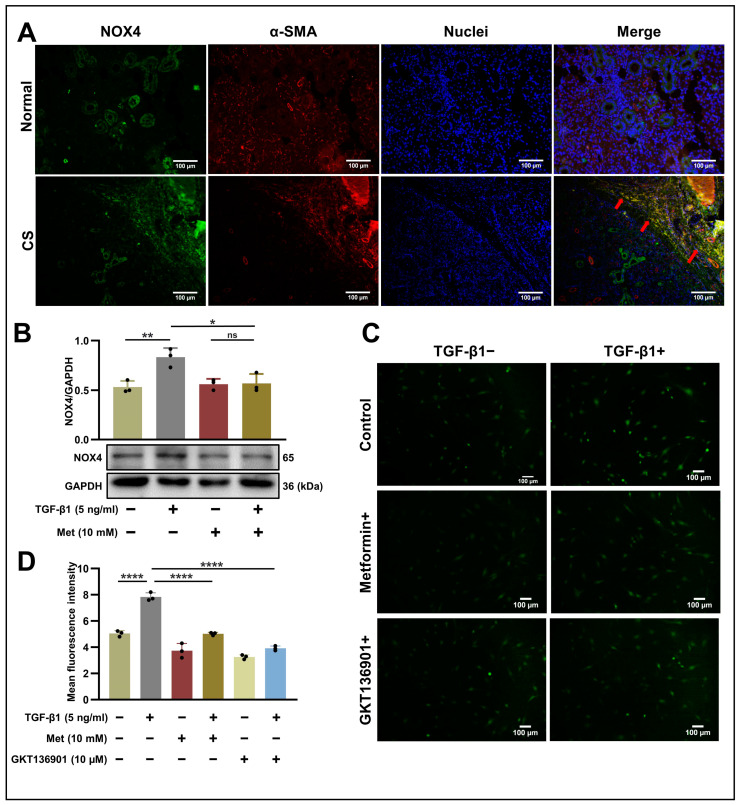
NOX4 participates in the fibrotic process by regulating ROS levels in HPSFs: (**A**) Representative immunofluorescence images displaying NOX4 (green), α-SMA (red), and nuclei (blue) in SMG sections from normal and CS tissues. Arrows indicate colocalization (scale bar: 100 µm). (**B**) Western blot analysis using anti-NOX4 and anti-GAPDH antibodies of cell lysates from control (lane 1, 2) and metformin (10 mM)-treated HPSFs (lane 3, 4). Metformin treatment commenced 24 h prior to TGF-β1 (5 ng/mL) stimulation, and protein samples were harvested following 48 h of TGF-β1 treatment. The upper panels display the mean (±SD) relative expression derived from three independent experiments. ns > 0.05, * *p* < 0.05, ** *p* < 0.01. (**C**,**D**) Fluorescence distribution of DCFH-DA staining for intracellular ROS production. Metformin and GKT136901 treatments were initiated 24 h before TGF-β1 (5 ng/mL) stimulation. After 20 min of incubation with DCFH-DA, DCF fluorescence was quantified using a fluorescence microscope. The right panels show the mean (±SD) relative expression from three independent experiments. **** *p* < 0.0001. HPSFs, human primary salivary gland fibroblasts; NOX4, NADPH oxidase 4; α-SMA, alpha-smooth muscle actin; CS, chronic sialadenitis; Met, metformin; TGF-β1, transforming growth factor-beta 1; GAPDH, glyceraldehyde-3-phosphate dehydrogenase; ROS, reactive oxygen species; SD, standard deviation; DCFH-DA, 2′,7′-dichlorofluorescin diacetate; DCF, 2′,7′-dichlorofluorescin.

Among the NOX family, NOX4 is most closely associated with fibroblasts and has been extensively investigated for its role in mediating ROS levels to affect the TGF-β1-SMAD2/3 signaling pathway [23]. In our study, IF staining of SMG tissue revealed that the expression levels of NOX4 and α-SMA in CS were markedly elevated, with partial colocalization of increased NOX4 and α-SMA at the collagen fiber deposition sites (Figure 3A). This suggests that NOX4 may be associated with myofibroblasts in the SMG. To clarify the role of NOX4 in the metformin-mediated inhibition of TGF-β1-activated HPSFs, we assessed the alterations in NOX4 protein expression levels in salivary gland fibroblasts following TGF-β1 treatment with or without metformin. The results indicated that TGF-β1 significantly upregulated NOX4 expression (*p* < 0.01), an effect that was counteracted by metformin (*p* < 0.05) (Figure 3B).

To further explore the role of ROS, we employed 2′,7′-dichlorofluorescin diacetate (DCFH-DA) to measure the ROS expression levels in cells treated with metformin and the NOX1/4 inhibitor, GKT136901. TGF-β1 treatment promoted ROS production (*p* < 0.0001), whereas metformin treatment diminished this effect (*p* < 0.0001). Similarly, GKT136901 also notably suppressed ROS production (*p* < 0.0001) in the presence of TGF-β1, confirming that NOX4 is involved in TGF-β1-induced ROS production (Figure 3C,D).

### 2.6. Metformin Reduces COL1A1 Production by Phosphorylating AMPK to Suppress NOX4-Mediated ROS in HPSFs

**Figure 4:**
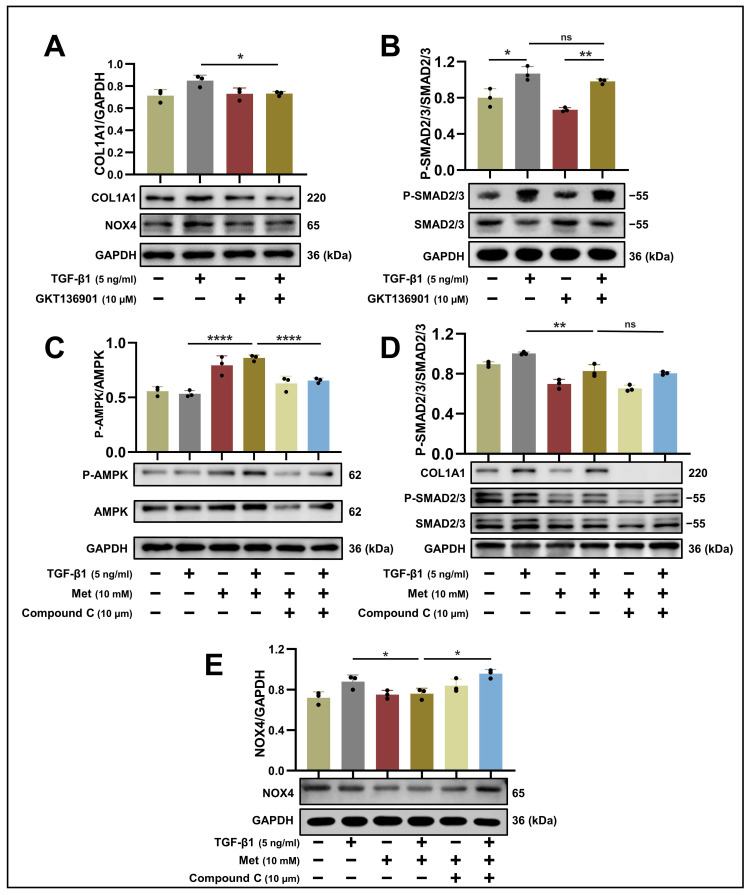
Metformin can influence NOX4 expression in TGF-β1-induced HPSFs by modulating AMPK: (**A**,**B**) Western blot analysis using antibodies against P-SMAD2/3, SMAD2/3, COL1A1, NOX4, and GAPDH of cell lysates from control (lane 1, 2) and GKT136901-treated HPSFs (lane 3, 4). GKT136901 treatment was initiated 24 h prior to TGF-β1 (5 ng/mL) stimulation, and protein samples were harvested following 48 h of TGF-β1 treatment. The upper panel displays the mean (±SD) of relative expression derived from three independent experiments. ns > 0.05, * *p* < 0.05, ** *p* < 0.01. (**C**–**E**) Western blot analysis using antibodies against phospho-AMPK, AMPK, NOX4, and GAPDH of cell lysates from control (lane 1, 2), metformin-treated (lane 3, 4), and Compound C-treated HPSFs (lane 5, 6). Metformin and Compound C treatments were initiated 24 h prior to TGF-β1 (5 ng/mL) stimulation, and protein samples were harvested after 48 h of TGF-β1 treatment. The upper panel shows the mean (±SD) of relative expression from three independent experiments. ns > 0.05, * *p* < 0.05, ** *p* < 0.01, **** *p* < 0.0001. HPSFs, human primary salivary gland fibroblasts; Met, metformin; TGF-β1, transforming growth factor-beta 1; NOX4, NADPH oxidase 4; COL1A1, collagen type I alpha 1; AMPK, adenosine 5′-monophosphate (AMP)-activated protein kinase; P-AMPK, phospho-AMPK; GAPDH, glyceraldehyde-3-phosphate dehydrogenase; SD, standard deviation.

To elucidate the interplay between NOX4 and the TGF-β1-SMAD2/3 signaling pathway, we assessed alterations in COL1A1 and P-SMAD2/3 protein expression levels in salivary gland fibroblasts following TGF-β1 treatment, with or without preconditioning with GKT136901. Our findings indicated that GKT136901 treatment led to a decline in COL1A1 levels (*p* < 0.05), whereas the phosphorylation status of SMAD2/3 remained largely unaltered (*p* > 0.05) (Figure 4A,B). However, metformin influenced SMAD phosphorylation levels, suggesting that NOX4 inhibition is only one component of metformin’s anti-fibrotic action. To further explore the role of NOX4 in the anti-fibrotic process of metformin, we also examined whether metformin could further enhance the anti-fibrotic effect in the presence of GKT136901. The results showed that in addition to GKT136901 inhibiting NOX4 expression and thereby producing an anti-fibrotic effect, metformin further inhibited fibrosis (Supplementary Figure S1B–D). This suggests that inhibition of NOX4 expression may only be one of the pathways through which metformin inhibits fibrosis, and metformin also exerts anti-fibrotic effects through other pathways.

While several AMPK-independent mechanisms of metformin have been unveiled in recent years, AMPK remains a principal effector of metformin’s therapeutic activity [24]. To explore the connection between AMPK and NOX4, HPSFs were exposed to the AMPK inhibitor, Compound C. This inhibitor was found to counteract the suppressive effect of metformin on NOX4 expression (*p* < 0.05) but not on SMAD2/3 (*p* > 0.05) (Figure 4C–E), implying that metformin may affect the expression of NOX4 through the regulation of AMPK.

### 2.7. Metformin Mitigates the Progression of Salivary Gland Fibrosis following Submandibular Gland Duct Ligation in Rats

To investigate the anti-fibrotic effects of metformin, we utilized rat models subjected to Wharton’s duct ligation, a known inducer of salivary gland fibrosis. Based on prior research [10], fibrotic responses were noted as early as one day following duct ligation, with pronounced fibrosis observable by day 12. Consequently, we initiated intragastric administration of metformin on day 1 post-ligation and selected day 12 as the time point for assessing the therapeutic impact on fibrosis.

**Figure 5:**
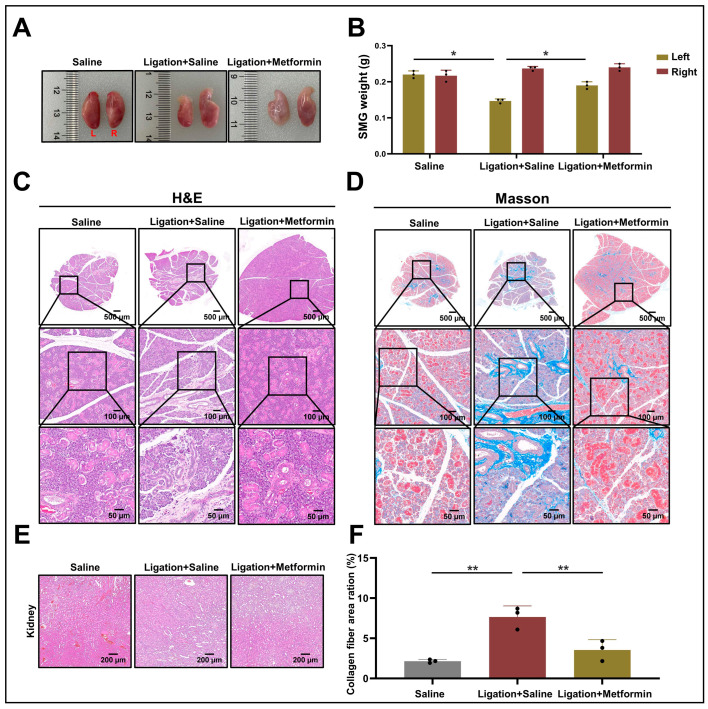
Effect of metformin on salivary gland fibrosis following submandibular gland duct ligation in rats: (**A**) Bilateral glands of rats. L: Left ligated SMG, R: Right non-ligated SMG (indicated in red text). (**B**) Weights of bilateral glands from the three experimental groups (*n* = 3). ns > 0.05, * *p* < 0.05. (**C**,**D**) H&E staining and Masson’s trichrome staining of the left SMG from each group. Middle panel images (scale bar: 100 µm) are enlargements of the regions indicated by boxes in the upper panels (scale bar: 500 µm), and the lower panels show further magnification (scale bar: 50 µm). (**E**) H&E staining of kidney tissues from each group (scale bar: 200 µm). (**F**) Quantitative expression of collagen fibers in left ligated SMG, * *p* < 0.05, ** *p* < 0.01. SMG, submandibular glands; H&E, hematoxylin and eosin.

Comparative analysis with the control group revealed significant glandular atrophy (*p* < 0.05) in the duct-ligation rats, an effect that was notably ameliorated in the metformin-treated group (*p* < 0.05) (Figure 5A,B). Histological examination employing both H&E staining and Masson’s trichrome staining further confirmed a substantial reduction in glandular fibrosis by day 12 in the metformin-treated group (*p* < 0.01), along with mitigation of acinar atrophy (Figure 5C,D,F). Additionally, given that metformin is primarily metabolized by the kidneys [25], we conducted H&E staining of kidney tissues from each experimental group and found no discernible pathological alterations, indicating that the drug’s toxicity at the administered concentration is within the acceptable limits (Figure 5E).

**Figure 6:**
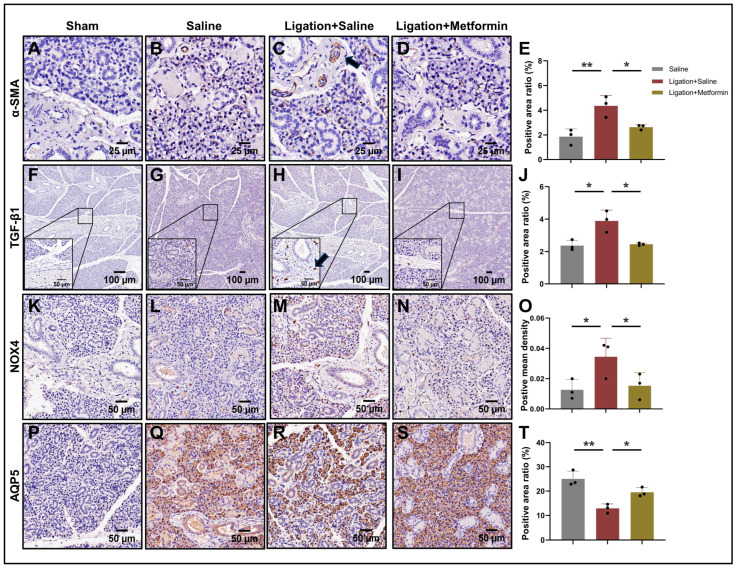
α-SMA, TGF-β1, NOX4, and AQP5 expression in left submandibular gland in rats: (**A**–**E**) α-SMA expression of rat SMG (scale bar: 25 µm). Arrows indicate positive expression, * *p* < 0.05, ** *p* < 0.01. (**F**–**J**) TGF-β1 expression of rat SMG (scale bar: 100 µm). Arrows indicate positive expression, * *p* < 0.05. (**K**–**O**) NOX4 expression of rat SMG (scale bar: 50 µm). * *p* < 0.05. (**P**–**T**) AQP5 expression of rat SMG (scale bar: 50 µm). * *p* < 0.05, ** *p* < 0.01. α-SMA, alpha-smooth muscle actin; TGF-β1, transforming growth factor-beta 1; NOX4, NADPH oxidase 4; AQP5, aquaporin 5.

Lastly, IHC analysis indicated that α-SMA (*p* < 0.05) and TGF-β1 (*p* < 0.05) expression levels were markedly decreased in the metformin-treated group, corroborating the in vitro findings (Figure 6A–J). We also assessed variations in NOX4 expression across the groups. Elevated levels of NOX4 were predominantly localized in regions of collagen fiber deposition surrounding the duct in the ligation group; however, these levels were modestly reduced following metformin treatment (*p* < 0.05) (Figure 6K–O). Intriguingly, a significant decline in the acinar marker aquaporin 5 (AQP5) was observed in the ligation group, whereas its expression nearly normalized in the metformin-treatment group (*p* < 0.05) (Figure 6P–T), suggesting a potential protective role for metformin on acinar cells.

## 3. Discussion

While the pathophysiological mechanisms underlying salivary gland fibrosis remain unclear, increasing evidence supports the notion that fibroblast differentiation into myofibroblasts and the subsequent secretion of large amounts of ECM by myofibroblasts are pivotal processes [26]. In this study, we collected clinical tissue specimens from patients and verified that both TGF-β1 (*p* < 0.01) and the myofibroblast marker α-SMA (*p* < 0.01) were significantly elevated in sialadenitis and highly enriched in the SMG tissue of fibrosis. Their levels correlated significantly with the degree of glandular fibrosis in the salivary gland.

Our findings also indicate that metformin inhibits salivary gland fibrosis by targeting and restraining the TGF-β1-SMAD2/3 signaling cascade. It is well established that the predominant signal transmission route for TGF-β1 is the extensively preserved SMAD pathway [27]. Crucial elements that influence the modulation of the TGF-β1-SMAD2/3 cascade encompass interactions between receptors, phosphorylation/dephosphorylation activated by receptors, interactions between SMAD proteins, transport of SMADs between the nucleus and cytoplasm, and the gathering of SMAD complexes that attach to distinct promoter regions to modify gene transcription. Upon TGF-β1 receptor stimulation, regulated SMADs, such as SMAD2 and SMAD3, undergo phosphorylation activation and dimerization and subsequently associate with Co-SMAD (SMAD4) to form heterotrimers. These activated SMAD assemblies are then relocated to the nucleus, where they cluster and associate with target gene sequences, thereby directly modulating the transcription of those genes [28,29]. Our study revealed that metformin reduced both SMAD2/3 phosphorylation levels (*p* < 0.01) and TGF-β1-induced COL1A1 levels (*p* < 0.01), which aligns with the previous findings of metformin alleviating pulmonary fibrosis [23]. At the same time, the observed inhibition of IL-1β (*p* < 0.01) by metformin appears to alter cellular conditions, potentially attenuating the function of TGF-β1. However, the precise nature of their interaction remains undefined and presents a promising avenue for further investigation.

Recent studies have revealed a relationship between NOX4-derived intracellular ROS levels and the TGF-SMAD2/3 pathway. NOX4 has been detected in fetal lung mesenchymal cells [15], lung fibroblasts [30], kidney fibroblasts [31], and hepatic stellate cells [32], where it regulates TGF-β1-mediated collagen expression. Each of these cell types is involved in the development of fibrosis in the affected organ. Moreover, ROS may play a regulatory role in activating the TGF-β1-SMAD2/3 pathway. For instance, in cardiac fibroblasts, TGF-β1-induced SMAD2/3 phosphorylation was significantly inhibited by various antioxidants [14]. In our study, the co-expression of NOX4 and α-SMA was elevated in specimens of chronic salivary gland inflammation. Subsequently, we found that metformin treatment affected NOX4 expression (*p* < 0.05), concurrent with a reduction in intracellular ROS levels. When HPSFs were treated with the NOX1/4 inhibitor GKT-136901, both COL1A1 levels (*p* < 0.05) and intracellular ROS were significantly reduced, although SMAD2/3 phosphorylation levels remained unchanged (*p* > 0.05).

Metformin’s well-documented pharmacological action involves the activation of AMPK [33]. In this study, cells were exposed to the AMPK inhibitor, Compound C, and it was observed that the suppression of NOX4 by metformin was counteracted, suggesting that metformin can influence the expression of NOX4 by regulating AMPK. However, Compound C did not significantly affect SMAD2/3 phosphorylation levels, indicating that metformin’s regulation of SMAD2/3 phosphorylation is AMPK-independent. This observation aligns with a study by Xiao et al. on the use of metformin to treat cardiac fibrosis [34].

Additionally, while metformin inhibited fibrosis and suppressed SMAD2/3 phosphorylation, NOX4 inhibitor anti-fibrosis action did not significantly affect SMAD2/3 phosphorylation levels. Therefore, we propose that metformin affects the generation of ROS by NOX4 through an AMPK-dependent mechanism and concurrently suppresses SMAD phosphorylation via an AMPK-independent mechanism, ultimately reducing the fibrotic effects induced by TGF-β1. However, the relationship between SMAD and NOX4 still requires further investigation. Notably, recent research by Howard E. Boudreau indicated that NOX4 transcriptional regulation by TGF-β1 is SMAD3-dependent and that NOX4 expression occurs downstream of SMAD2/3 activation [11].

The Wharton’s duct ligation-induced salivary gland injury model is commonly used in studies of salivary gland diseases. This process typically involves collagen accumulation, progressive fibrosis, and gland remodeling [35]. However, the mechanism of resolution remains unclear. We investigated whether metformin could alleviate Wharton’s duct ligation-induced salivary gland fibrosis by inhibiting the TGF-β1-SMAD2/3 pathway. Rats received daily metformin doses (100 mg/kg) via oral gavage. Although the dose used in this study exceeded the typical dosage for patients with diabetes (10–40 mg/kg), past research assessing the anti-diabetic and anti-cancer properties of metformin in murine models have employed increased dosages (250–350 mg/kg) due to the variability in drug responsiveness between rodents and humans [36,37]. Consequently, we contend that a daily dose of 100 mg/kg of metformin is permissible in a murine model.

On day 12, histology and IHC staining revealed significant reductions in collagen and pro-fibrotic biomarkers, including α-SMA and TGF-β1. Additionally, we observed a significant decrease in NOX4 levels (*p* < 0.05) after metformin treatment, consistent with the results of our in vitro experiments. This finding confirms the important role of NOX4 in inhibiting fibrosis. It is important to note that the precise concentration of metformin required for anti-fibrotic effects in the rat submandibular gland, when administered orally, remains elusive. Additionally, whether the mechanism of action at this concentration is fully consistent with in vitro observations warrants further investigation.

Significantly, our study revealed that metformin treatment leads to the acinar marker AQP5 approaching normal levels (*p* < 0.05), suggesting that metformin has a dual role in acinar protection as well as anti-fibrosis, although the precise mechanism of action warrants further investigation. The therapeutic advantages may largely be attributed to metformin’s action on cells beyond fibroblasts. Ji-Won Kim suggested that metformin may influence a variety of cells, offering potential therapeutic benefits for both humans and animals [38]. Previous studies have also indicated that metformin can suppress the generation of ROS by stimulating autophagy, consequently shielding cells from damage induced by oxidative stress [39]. Additionally, metformin has been demonstrated to safeguard endothelial cells by activating the suppressed autophagy via the Hedgehog (Hh) pathway under conditions of hyperglycemia [40]. Thus, it is reasonable to hypothesize that the acinar-protective effect of metformin could be associated with the modulation of autophagy levels.

Our study presents pioneering evidence that metformin treatment results in a marked reduction in glandular fibrosis, which is mediated by the inhibition of SMAD2/3 phosphorylation in an AMPK-independent manner and the regulation of NOX4 expression in an AMPK-dependent manner. Furthermore, the protective influence of metformin on acinar cells holds significant implications. It is worth noting that in our cell experiment, metformin was pretreated for 24 h, which may have caused a subtle change in the cellular state compared to direct treatment. This alteration could also play a role in the anti-fibrotic action of metformin. However, while this preliminary investigation into metformin’s potential against salivary gland fibrosis provides promising insights, it is not without limitations. Future research might benefit from examining the interaction between NOX4 and SMAD2/3 and AMPK’s impact on SMAD2/3 phosphorylation, which may be mediated in a metformin concentration-dependent manner. Expanding the study to a larger sample size of KO animal experiments could further elucidate metformin’s in vivo anti-fibrosis mechanisms.

In conclusion, our findings affirm metformin’s therapeutic potential against salivary gland fibrosis. These insights may shed light on innovative therapeutic strategies for fibrotic diseases of the salivary gland, potentially positioning metformin as a viable treatment option. Nonetheless, further trials are essential to validate crucial considerations, including pathway regulation mechanisms and the drug’s clinical efficacy.

## 4. Materials and Methods

### 4.1. Human SMG Tissues

A total of ten human SMG tissue samples, which included five cases of sialadenitis and five normal specimens, were obtained from the Pathology Department at the School and Hospital of Stomatology, Wuhan University. The normal samples were collected from SMG tissues procured during neck dissection surgeries for maxillofacial tumors and were verified as normal through histological examination. Sialadenitis specimens exhibited mild to moderate inflammation upon histological examination, while cases of severe sialadenitis were excluded due to the absence of essential histological structures.

### 4.2. Histological Staining

Human and rat SMG tissues were fixed in paraformaldehyde, embedded in paraffin, and sectioned into 4 μm slices. Histological alterations were visualized using H&E (#G1076, Servicebio, Wuhan, China) staining, while fibrosis was assessed via Masson’s trichrome (#G1006, Servicebio, Wuhan, China) and Sirius red (#GC307014, Servicebio, Wuhan, China) staining as per manufacturers’ protocols. ImageJ software (ImageJ, National Institutes of Health, Bethesda, MA, USA) facilitated the semi-quantitative analysis of glandular morphological changes in a blinded manner.

### 4.3. Immunohistochemistry (IHC) Staining

IHC was executed as per a previously established protocol [41]. In summary, the sections underwent deparaffinization in xylene, rehydration in a series of graded alcohol, and antigen retrieval was performed using citrate buffer (pH 6.0). Subsequently, the sections were treated with a blocker for endogenous peroxidase and goat serum at 37 °C for a duration of 20 min, followed by incubation at 4 °C with primary antibodies directed against TGF-β1 (#21898-1-AP, 1:400, Proteintech, Wuhan, China), α-SMA (#MA5-11547, 1:400, Thermo Fisher Scientific, Waltham, MA, USA), NOX4 (#A11274, 1:100, ABclonal, Wuhan, China), and AQP5 (#ab78486, 5 μg/mL, Abcam, Cambridge, UK) overnight. Subsequently, sections were incubated with a biotinylated secondary antibody and anti-biotin–peroxidase reagent at 37 °C for 20 min. Diaminobenzidine (DAB) substrate (MXB Biotechnologies, Fuzhou, China) was employed for staining visualization. All samples were treated with DAB substrate for the same duration, counterstained with hematoxylin, and scanned using an Aperio ScanScope CS scanner (Aperio, Sausalito, CA, USA). For semi-quantitative analysis using ImageJ, four random fields of view were chosen. The positive area was designated as a representative α-SMA, TGF-β, and AQP5 staining area (indicating the relative α-SMA, TGF-β, and AQP5 expression level). The mean densitometry of the digital image was designated as representative NOX4 staining intensity (indicating the relative NOX4 expression level). The positive area and the mean densitometry of the tissue areas from five randomly selected fields were counted in a blinded manner and subjected to statistical analysis.

### 4.4. Immunofluorescence (IF) Staining

Cells or tissue sections underwent IF staining, as outlined before [41]. The cells were treated with 4% paraformaldehyde for fixation, followed by permeabilization using 0.2% Triton X-100, and then blocked with 5% bovine serum albumin for one hour. For tissue sections, the processes of antigen retrieval and blocking of endogenous peroxidase were executed as specified in the IHC section. Subsequently, cells or sections were left to incubate at 4 °C overnight with primary antibodies targeting NOX4 (#A11274, 1:100, ABclonal, Wuhan, China), α-SMA (#MA5-11547, 1:250, Thermo Fisher Scientific, Waltham, MA, USA), and vimentin (#60330-1-Ig, 1:500, Proteintech, Wuhan, China), followed by a 1 h incubation with fluorophore-conjugated secondary antibodies (Abmart, Shanghai, China). Nuclei were stained with DAPI (Beyotime, Shanghai, China), and images were captured using a Zeiss LSM880 Fast microscope and ZEN 2.3 Software (Zeiss, Jena, Germany).

### 4.5. Western Blot Analysis

The process of Western blotting was executed as detailed earlier [42]. Cells were lysed using Radio Immunoprecipitation Assay (RIPA) Lysis buffer (Beyotime, Shanghai, China) containing 1 mM phenylmethanesulfonyl fluoride (PMSF) (Servicebio, Wuhan, China) and phosphatase inhibitor cocktail A (Beyotime, Shanghai, China) on ice. Protein from the cell lysates was then quantified and equalized before undergoing separation on a 10% sodium dodecyl sulfate (SDS)–polyacrylamide gel and then transferred onto polyvinylidene difluoride (PVDF) membranes (#IPVH00010, Millipore, Burlington, MA, USA). Following this, the membranes were blocked using 5% skim milk and then left to incubate at 4 °C overnight with primary antibodies against α-SMA and COL1A1 (#19245, 1:1000 and #72026, 1:1000, Cell Signaling Technology, Danvers, MA, USA), NOX4, SMAD2/3, and phospho-SMAD2/3 (#A11274, 1:1000, #A18674, 1:500 and #AP0548, 1:500, ABclonal, Wuhan, China); AMPK and phospho-AMPK (#ab207442, 1:1000 and #ab133448, 1:1000, Abcam, Cambridge, UK); and IL-1β and GAPDH (#GB11113-100, 1:1000, and #GB11002-100, 1:1000, Servicebio, Wuhan, China). After several washes with Tris Buffered Saline with Tween 20 (TBST) (Servicebio, Wuhan, China), membranes were incubated with secondary antibodies (#AS003 and #AS014, 1:5000, ABclonal, Wuhan, China) and visualized using an ECL kit (#K-12045, Advansta, San Jose, CA, USA) and the Odyssey system (LI-COR Biosciences, Hainesport, NJ, USA). ImageJ software was used for image analysis. The researchers for immunoblot signals measuring were blinded to any grouping information.

### 4.6. Cell Culture

HPSFs were isolated using the tissue explant method. Normal salivary gland tissues were obtained from two male and one female patients (aged 30–60 years) with maxillofacial neoplasms who underwent submaxillary gland removal via neck dissection. As previously described [43], fresh submandibular gland tissues were diced into individual cubes, placed in a cell culture flask, and maintained in Dulbecco’s modified Eagle medium (DMEM) (#C11995500BT, Thermo Fisher Scientific, Waltham, MA, USA) enriched with 100 U/mL of penicillin, 100 µg/mL of streptomycin, and 10% of fetal bovine serum (FBS) (#A2437266, Gibco, Waltham, MA, USA). The cells were then cultured in an incubator set at 37 °C and 5% CO_2_. HPSFs were characterized by examining the expression of mesenchymal marker vimentin. HPSFs from each donor were used independently for experiments. Cells from the fourth to sixth passages were utilized for all analyses. Recombinant human TGF-β1 was acquired from Peprotech (#100-21, Cranbury, NJ, USA), metformin from Biogems (#1117045, Cranbury, NJ, USA), and Compound C (#HY-13418A) and GKT136901 (#HY-101499) from MCE (Monmouth Junction, NJ, USA). Metformin, Compound C, and GKT136901 pretreated cells 24 h before TGF-β1 stimulated cells.

### 4.7. Cellular Reactive Oxygen Species Measurement

HPSFs were seeded into 6-well plates, maintaining a density of 1.2 × 10^6^ cells for each well (# 140675, Thermo Fisher Scientific, Waltham, MA, USA). The total cellular ROS was assessed using a ROS Assay Kit (#S0033S, Beyotime, Shanghai, China), as per the instructions provided by the manufacturer. Cells were treated with 10 μM of DCFH-DA at a temperature of 37 °C for a duration of 20 min, followed by three washes using a serum-free medium. The DCF fluorescence was then observed using a fluorescence microscope (Leica, Wetzlar, Germany), with the excitation and emission wavelength set at 488 nm and 525 nm, respectively.

### 4.8. Animal Model

Nine healthy female Wistar rats weighing 180–220 g were randomly assigned to control, ligation, and administration groups, with three rats in each group. The ligation and administration groups underwent Wharton’s duct ligation as previously described [10]. From the day after surgery, the administration group received metformin at a dose of 100 mg/kg via intragastric administration, while the control and ligation groups received an equivalent volume of normal saline every 24 h. Body weight was recorded daily. Key step images are provided in Supplementary Figure S2. On the 12th day, all rats were euthanized, bilateral submandibular glands were excised and weighed, and then the left submandibular gland was bisected. One portion was fixed with 4% paraformaldehyde, dehydrated, embedded in paraffin, and sectioned. The other portion was frozen at −80 °C for protein analysis. Finally, the rat kidneys were extracted for fixation and embedding.

### 4.9. Statistical Analysis

Quantitative data are expressed as mean ± standard deviation. At least three independent experimental results were included in the analysis. All quantitative data are blindly processed before being handed to the analyst. The Student’s *t*-test was used for pairwise comparisons, and one-way ANOVA was used for multiple comparisons. Two-factor ANOVA was employed to analyze glandular weight comparisons. The threshold for statistical significance was set at *p* < 0.05. Prism v.9 (GraphPad Software, Inc., San Diego, CA, USA) was used for statistical analysis.

## Data Availability

The data presented in this study are available on request from the corresponding authors.

## Supplementary Materials

The following supporting information can be downloaded at: https://www.mdpi.com/article/10.3390/ijms242216260/s1.
