# Supplementary material for: Metformin Attenuates TGF-β1-Induced Fibrosis in Salivary Gland: A Preliminary Study
Source: Int J Mol Sci. 2023 Nov 13;24(22):16260. doi: 10.3390/ijms242216260 (PMC10671059; doi:10.3390/ijms242216260)
Supplement: Supplementary file 1 [file ijms-24-16260-s001.zip › ijms-2611346-Supplementary Figures/ijms-2611346-Supplementary Figures.pdf]

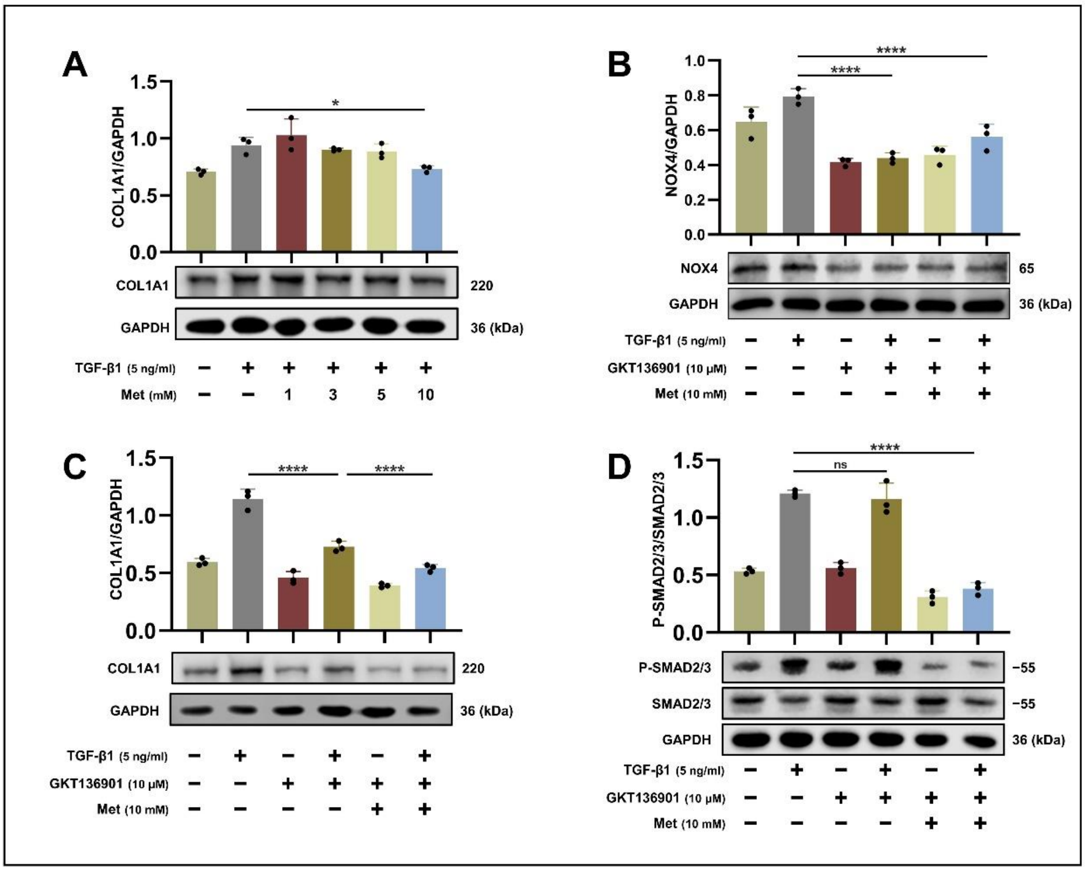

Supplementary Figure S1. Metformin attenuates the fibrotic effects of TGF-β1 in HPSFs. (**A**) Fibroblasts were exposed to TGF-β1 and metformin, and cell lysates were analyzed via SDS-PAGE and Western blot analysis for COL1A1 and GAPDH. The 10mM concentration was chosen as the effective inhibitory concentration of metformin for type I collagen production (n = 3 per group). *P < 0.05. (**B-D**) Western blot analysis using anti-COL1A1, anti-SMAD2/3, anti-phospho-SMAD2/3 (P-SMAD2/3), anti-NOX4 and anti-GAPDH antibodies on cell lysates from control (lane 1, 2), 10 μM GKT136901 (lane 3, 4) and 10 mM Metformin (lane 5, 6) treated HPSFs. Metformin treatment began 24h before TGF-β1 (5 ng/ml) stimulation, and protein samples were collected after 48 h of TGF-β1 treatment. Upper panels show the average (±SD) relative expression from three independent experiments. ns > 0.05, ***P < 0.001, ****P < 0.0001. Met, metformin; TGF-β1, transforming growth factor-beta 1; COL1A1, collagen type I alpha 1; NOX4, NADPH oxidase 4; GAPDH, glyceraldehyde-3-phosphate dehydrogenase; SD, standard deviation.

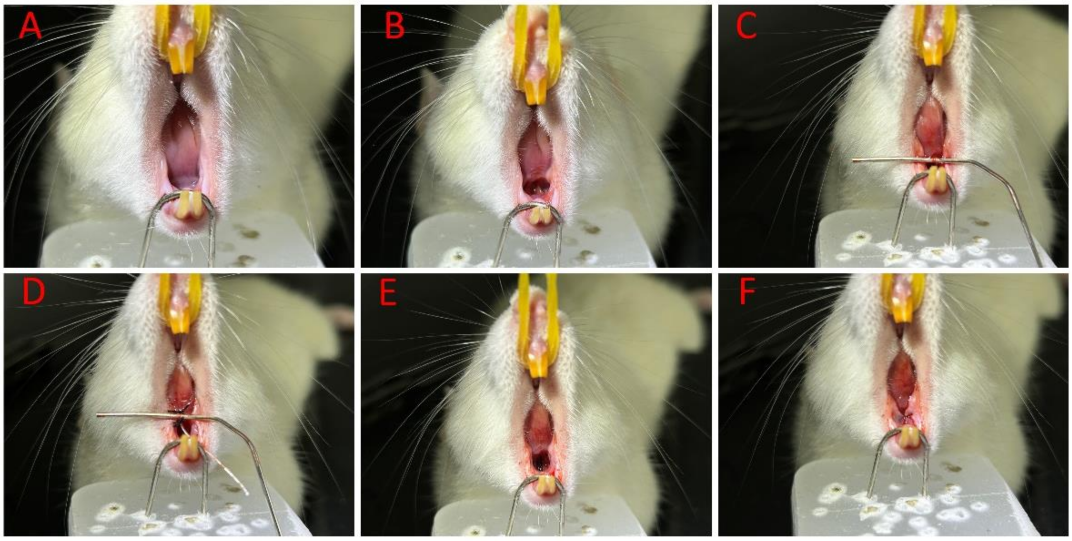

Supplementary Figure S2. Ligation of the Wharton's duct in rats. (**A**) Intra-oral view of the sublingual region in rats. (**B**) Slight separation of the mucosa. (**C**) Utilization of a blunt probe to identify the anterior portion of the duct. (**D**) A fish line with a diameter of 0.33 mm, positioned parallel to the duct, was secured using a 5–0 silk thread approximately 5 mm posterior to the ductal orifice before its removal. Subsequently, a second ligation was performed roughly 3 mm from the proximal end of the initial ligation site using the same technique, resulting in a double ligation. (**E**) Intra-oral view post-operation. (F) Mucosal suturing.
