# Supplementary material for: Metformin Attenuates TGF-β1-Induced Fibrosis in Salivary Gland: A Preliminary Study
Source: Int J Mol Sci. 2023 Nov 13;24(22):16260. doi: 10.3390/ijms242216260 (PMC10671059; doi:10.3390/ijms242216260)
Supplement: Supplementary file 1 [file Response to Reviewers.pdf › ijms-2611346-Supplementary Figures/Response to Reviewers.pdf]

Reviewers’ comments with the Authors’ responses

Round 1:

Response to Reviewer 1’s comments:

Dear reviewer,

Thank you for taking the time to review our research. We are glad that you acknowledged our efforts in exploring metformin's anti-fibrotic properties We value your feedback and are committed to addressing any concerns you've raised.

Lianhao Wang et al., in this manuscript explored metformin’s anti-fibrotic properties through human samples, primary salivary gland fibroblasts, and a rat model by a series of experimental evidence (histological (H&E and MT, confocal and Immunohistochemical Staining), and Wb. Precedents have been described in the use of metformin in various pathologies in this context. However, in this pathology, it is not

All the methodology and the evidence shown here are very interesting and relevant, however, there are some minor points corrections.
It seems that given the timing of the metformin treatment is for prevention, it would be interesting to see the expression of at least one interleukin (inflammation (PCR or Wb))

Response: Thank you for highlighting the importance of interleukin expression in relation to our study. We concur that evaluating the expression of interleukins can provide additional insights. As indicated in a literature source (PMCID: PMC2774671), IL-1β amplifies the profibrotic effects of TGF-β. Our WB experiments have indeed shown that metformin treatment inhibits IL-1β production, as depicted in **revised Figure 2F**. We hypothesize that the observed reduction in type I collagen might be linked to this inhibition of IL-1β. We plan to delve deeper into this mechanism in our future research.

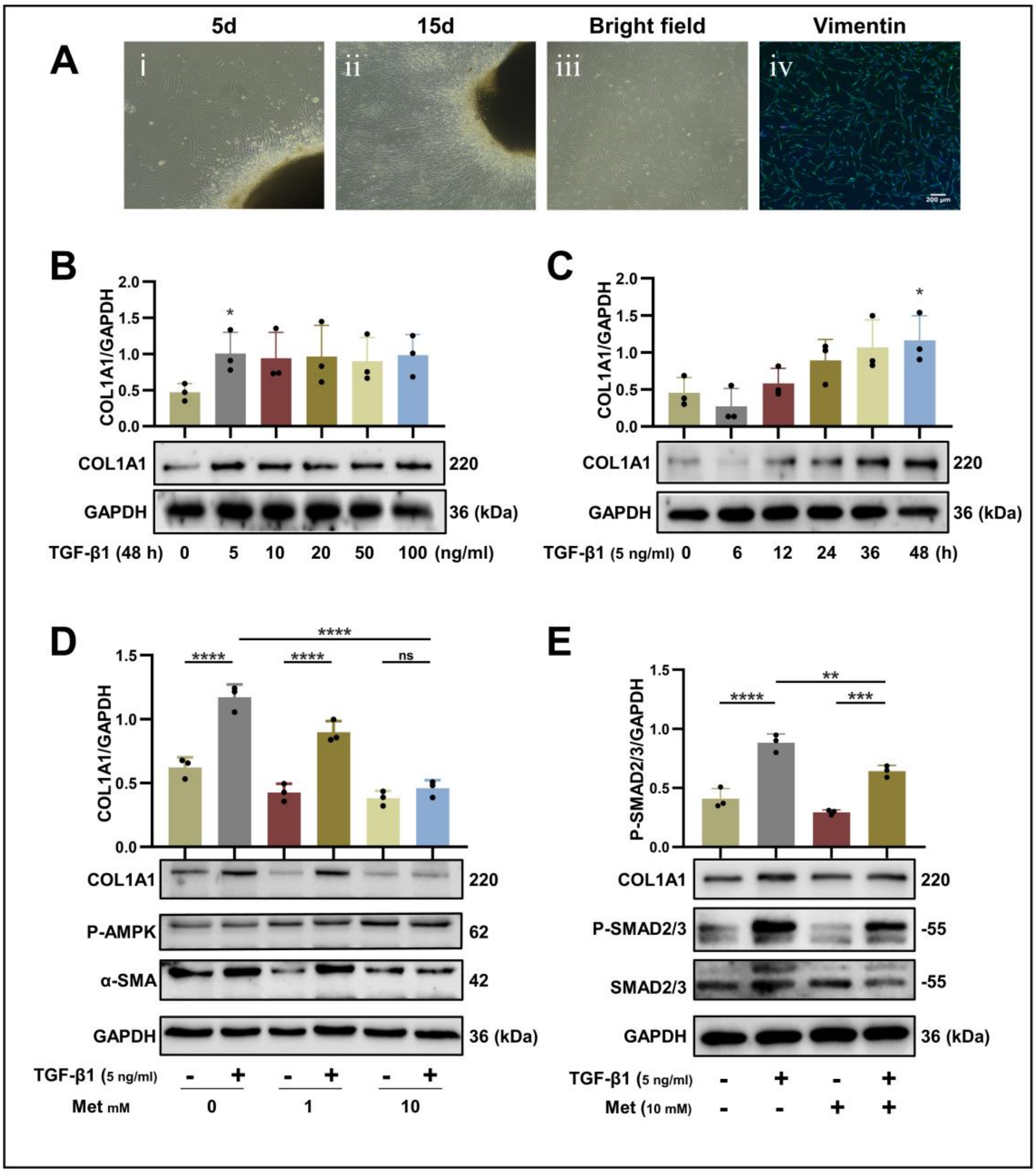

Original Figure 2

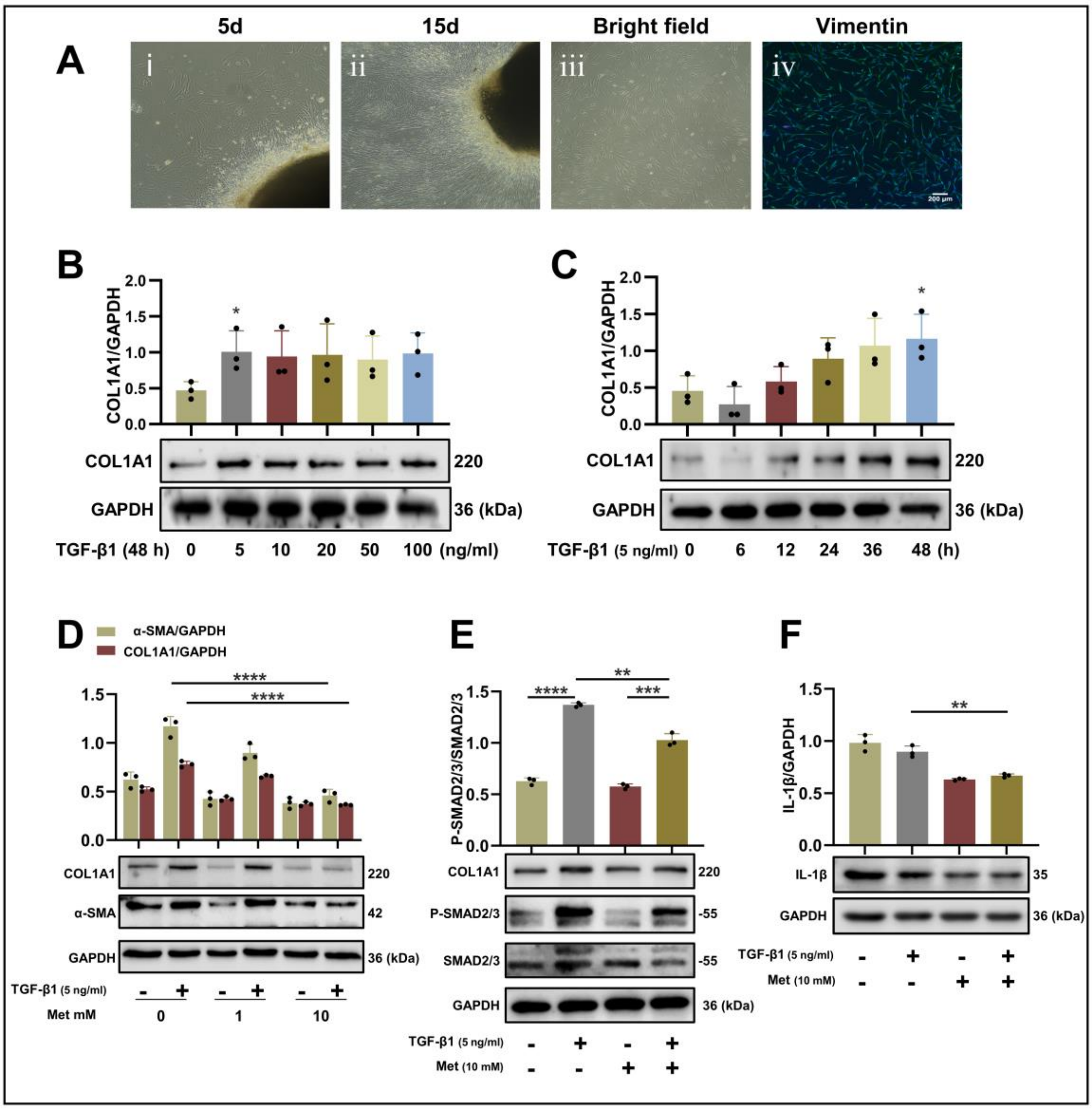

Revised Figure 2

The authors, in Figure 2d do not mention p-ampk in the text, they should show the total AMPK Wb.

Response: Thank you for pointing out the discrepancy regarding **original Figure 2D**. We acknowledge the importance of showing total AMPK WB for a comprehensive understanding. However, upon further reflection, we realized that **Figure 2**'s focus is on the phenotypic indicators, whereas AMPK serves as a mechanistic indicator. To maintain the coherence and logical structure of the article, we have chosen to remove the P-AMPK indicator. We appreciate your feedback and will ensure clarity in our revised manuscript.

Antibody concentration

Response: Thank you for raising the point about antibody concentrations. The concentrations of all antibodies used have been explicitly detailed in the **Methods** section of our manuscript.

Authors must specify the time of metformin treatment in the cells (methodology)

Response: Thank you for pointing out the omission regarding the timing of metformin treatment in our methodology. We've updated our manuscript to clearly specify the administration times of metformin, Compound C, and GKT136901 in the "**4.6. Cell Culture**" section of the **Methods**. Additionally, we have elaborated on this in the figure legends for **Figures 2, 3, and 4** for better clarity. We appreciate your keen observation and feedback.

We deeply value your expert guidance which has been instrumental in refining our work. Your time and expertise are genuinely appreciated. Should there be further areas of improvement in our manuscript, we warmly welcome your suggestions.

Response to Reviewer 2's comments:

Wang et al propose that pathologic fibrosis in salivary glands is driven by increased TGFb1 signaling and that, conversely, treatment with the diabetes medication Metformin can attenuate TGFb1-induced fibrosis via AMPK-dependent and -independent mechanisms. The authors thus suggest Metformin as a potential treatment in settings of salivary gland fibrosis.

A strength of the study is the suitable combination of cell culture work, an animal study, and the analysis of patient samples to corroborate the overall hypothesis. The manuscript is generally well-organized and -written. In confirmation of the overall idea, Metformin has already been shown to exert anti-fibrotic effects in various model systems, but its application to salivary gland fibrosis in the present study is somewhat novel.

However, due to several experimental/technical issues (e.g. poor quality blots, unsuitable choice of drug dose and timing, missing treatment groups) I am not convinced that the mechanistic insights derived from the cell culture work (Fig. 6) actually explain the anti-fibrotic effects of Metformin in vivo, thus lowering my enthusiasms for this study.

Dear Reviewer,

Thank you for your comprehensive review and thoughtful feedback on our research. We genuinely value your recognition of the novel application of metformin to salivary gland fibrosis and the strength of our multi-modal research approach.

We acknowledge the experimental and technical concerns you raised, particularly regarding the quality of blots, drug dosage, timing, and missing treatment groups. We agree that these issues warrant further scrutiny to ensure that our mechanistic insights from cell culture work genuinely resonate with the *in vivo* anti-fibrotic effects of metformin.

Your feedback provides essential guidance, and we are committed to addressing these concerns to bolster the integrity and impact of our study.

Specific points:

Ln354-362: The authors properly justify using a somewhat higher dose of Metfomin in their rat model (100 mg/kg), compared to doses used in T2D patients (40 mg/kg). However, they miss the larger point that the effective doses of Metformin upon treatment in humans or their rat model are orders of magnitude lower (serum levels are likely <50 microM at peak and <1 microM at steady-state) compared to the high doses of Metformin (10 milliM) used in cell culture experiments. This makes it likely that Metformin acts in vivo by mechanisms entirely distinct from those explored/proposed in cell culture. Doses of 10mM Metformin are more relevant to killing cancer cells in culture.

Response: We appreciate your thoughtful feedback regarding the significant disparity between the *in vivo* and *in vitro* dosages of metformin used in our study.

You rightly pointed out the stark difference between the serum levels likely achieved in the rat model and the considerably higher concentration used in cell culture experiments. This raises valid concerns about potential variations in the mechanisms of action between *in vivo* and *in vitro* settings.

The concentration of metformin in the rat submaxillary gland is influenced by several factors, including the method of administration, duration, and dosage. While there is limited literature regarding the absolute bioavailability of metformin in rodent species, no studies to our knowledge have explored its pharmacokinetics specifically in the salivary gland. Recent research into the application of metformin for treating radiation-induced salivary gland damage employed a concentration of 100mg/kg (PMID: 36726289). Given concerns about drug toxicity, our study also adopted a conservative approach, utilizing the same 100mg/kg concentration.

For our *in vitro* experiments, we selected a concentration of 10mM, driven by its observed inhibitory effect on COL1A1 as presented in **revised Figure 2D**. This concentration aligns with those used in prior studies investigating metformin's antifibrotic properties across various organs such as the lung, kidney, liver, and nose

(PMID: 27576730, PMID: 25744403, PMID: 21567395, PMID: 24357397). It's worth noting that variations in dosage selection across studies might arise from differing cellular sensitivities to metformin or potential inconsistencies in drug concentrations provided by various manufacturers.

Nevertheless, your feedback underscores the need for a more nuanced approach in determining the optimal concentration that aligns with both *in vitro* and *in vivo* experiments. This will indeed be a primary focus in our subsequent investigations, ensuring that our findings are both robust and translational.

Thank you once again for your insightful critique, which will undoubtedly guide our future endeavors.

Determining concentration-response curves for all readouts represents a missed opportunity that could allow the authors to confirm or disprove their proposed mechanisms. For example, if the same doses of Metformin are required to produce various readouts (e.g. reduction in COL, P-SMAD, P-AMPK) it would suggest that they are indeed causally related. Conversely, the drug's AMPK-dependent and AMPK-independent effects may be mediated by distinct effectors and hence by very distinct concentrations of Metformin (Fig. 6).

Response: Thank you for highlighting the importance of concentration-response curves to discern the mechanisms of metformin's action. Our study serves as an initial exploration into the impact of metformin on salivary gland fibrosis. Our primary objective was to discern the potential anti-fibrotic effects of metformin and elucidate the underlying mechanisms. After determining an effective inhibitory concentration from our findings (**revised Figure 2D**), our focus shifted to understanding the associated mechanistic pathways at this specific concentration. However, we have yet to examine the relationships between varying concentrations of metformin and the changes in pathway indicators. Delving deeper into these relationships in future experiments will offer a more comprehensive understanding of metformin's anti-fibrotic effects.

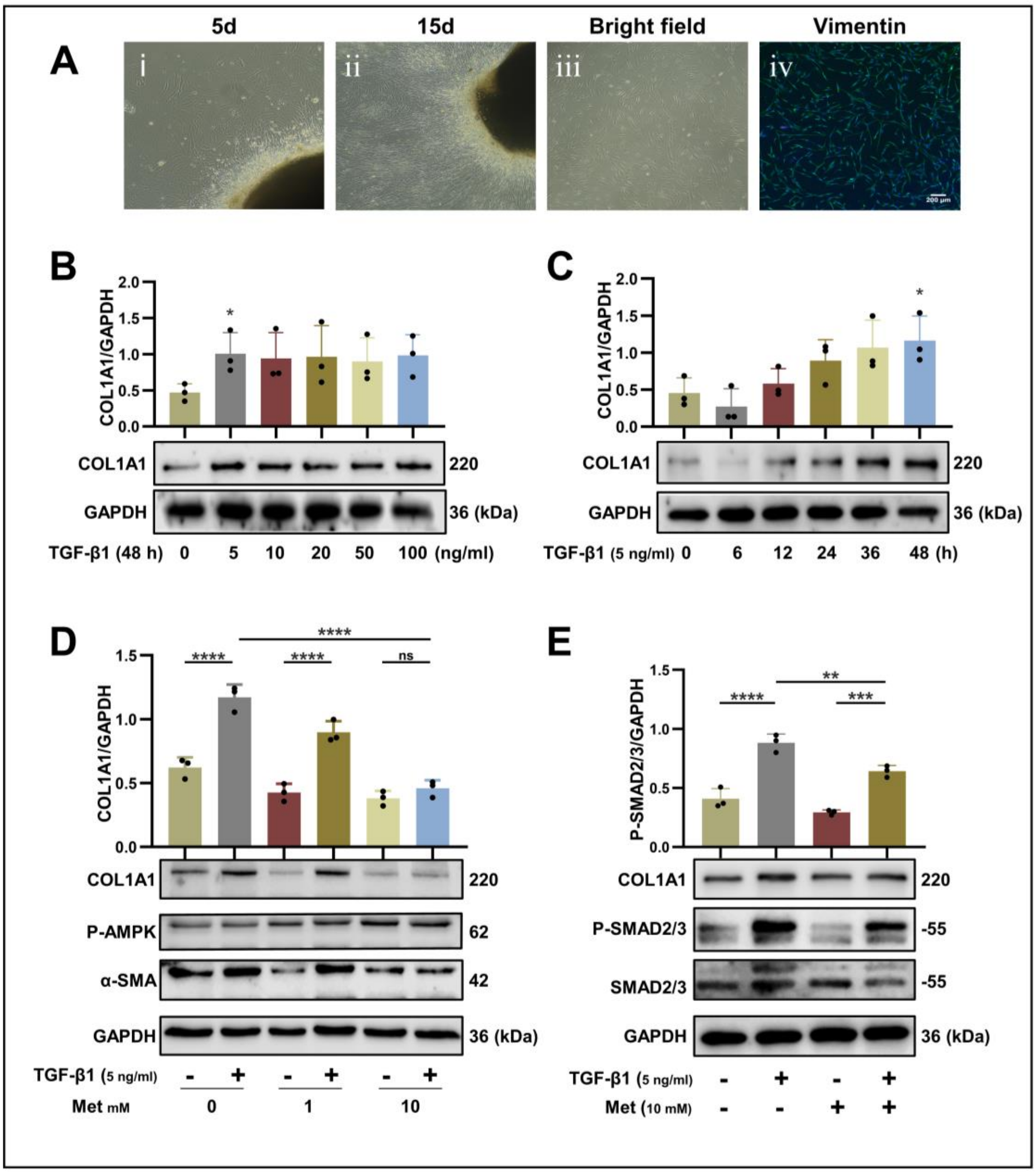

Original Figure 2

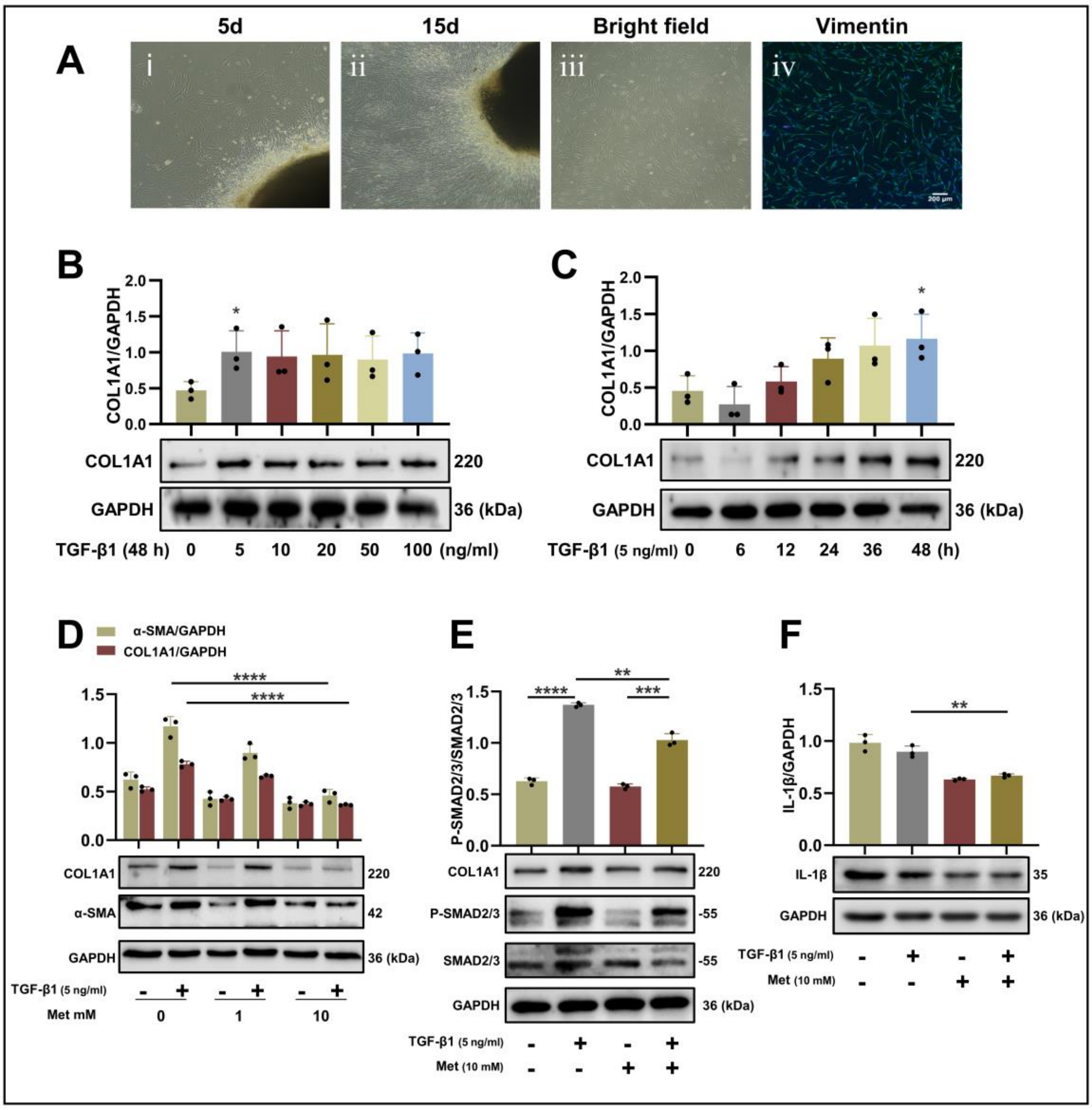

Revised Figure 2

The authors propose that Metformin exerts its effects by attenuating signaling events induced by the addition of TGFb to their cell cultures (Fig. 6). If so, it is unclear why cultures are pre-treated with Metformin 24 h before the addition of TGFb throughout all experiments. If this pre-treatment is necessary to produce Metformin's benefits, it would suggest that Metformin acts by rewiring the cellular environment prior to TGFb treatment, rather than modulating acute TGFb signaling as suggested by Fig. 6.

Response: We appreciate your keen observations and the concerns raised about our methodology. Our initial representation in **original Figure 6** aimed to succinctly illustrate the mechanism being investigated. However, to ensure clarity and avoid potential misconceptions, we've opted to omit this figure.

Our choice of pre-treatment with metformin was informed by preliminary literature findings, wherein most anti-fibrosis effects of metformin were observed through pre-treatment. During the course of our experiments, it was also found that simultaneous treatment of cells with TGF-β and metformin, without any prior pre-treatment, yielded results comparable to the pre-treatment approach.

Nevertheless, the intricacies of metformin's action are multifaceted and not entirely captured by **original Figure 6**. Our intention was to present the findings in a manner most comprehensible to our readers. Future studies will delve deeper into these nuances, and we are grateful for your feedback in steering our approach.

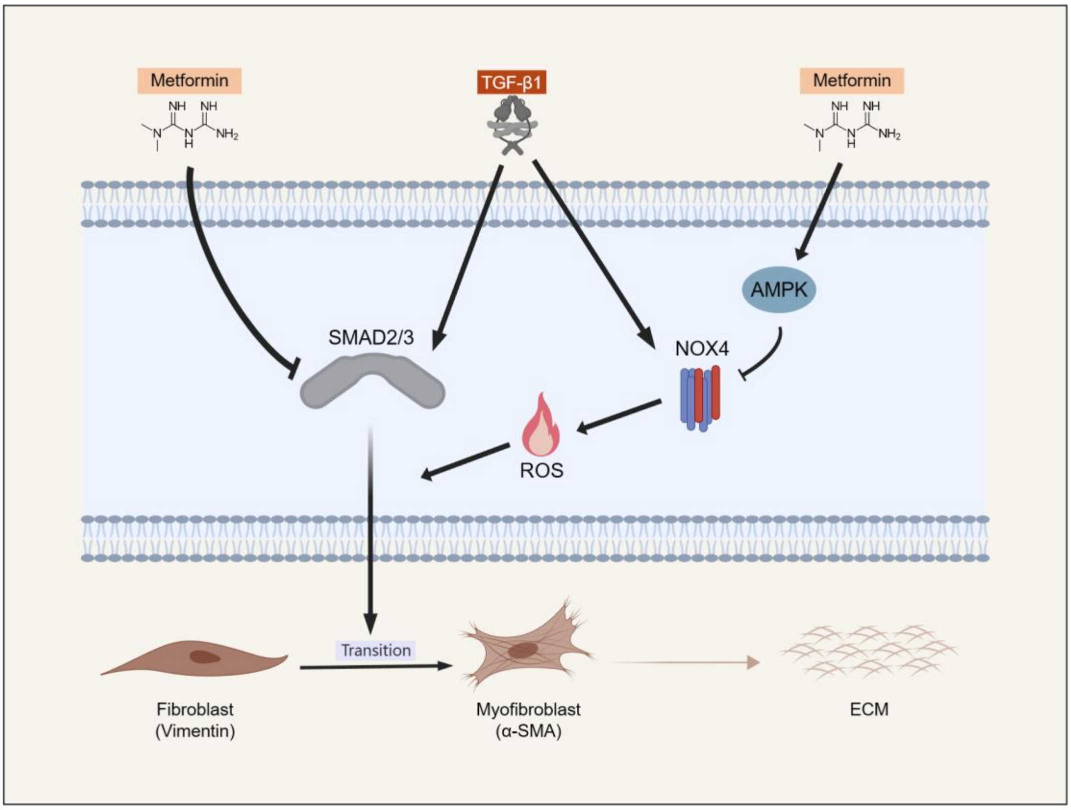

Original Figure 6

When using drugs to delineate the molecular pathways of Metformin action, the authors should always test each drug by itself, and then in combination with Metformin, to confirm the reported effects are NOT co-incidence, but causality. For example, in Fig. 3D/and 4A/B, the authors report the effect of Metformin or GKT136901 to suggest that Metformin acts via NOX. If this is the case, then Metformin should have no further effects on these readouts (e.g. ROS) in the presence of GKT136901, a treatment group (Metformin+GKT) that the authors did not test. In a similar vein, in Fig. 4C/D/E, the authors propose a role for AMPK by studying the effects of Metformin in the presence of Compound C, but miss to explore/report whether Compound C exerts effects by itself, and thus perhaps just shifts the baseline.

Response: We sincerely appreciate your insights, which greatly enhance the rigor of our study. Your point regarding the potential conflation of coincidence with causality in drug interactions is well-taken.

In **Figure 3D** and **revised Figure 4A/B**, while GKT136901 is recognized as an inhibitor of NOX4 known to regulate fibrosis by attenuating ROS production, we acknowledge the need to more definitively establish the interrelation between metformin and NOX4. Although our findings suggest that metformin's anti-fibrotic effect might be associated with NOX4 expression, the direct link warrants further investigation. This gap in our research is transparently discussed in the limitations section of our study.

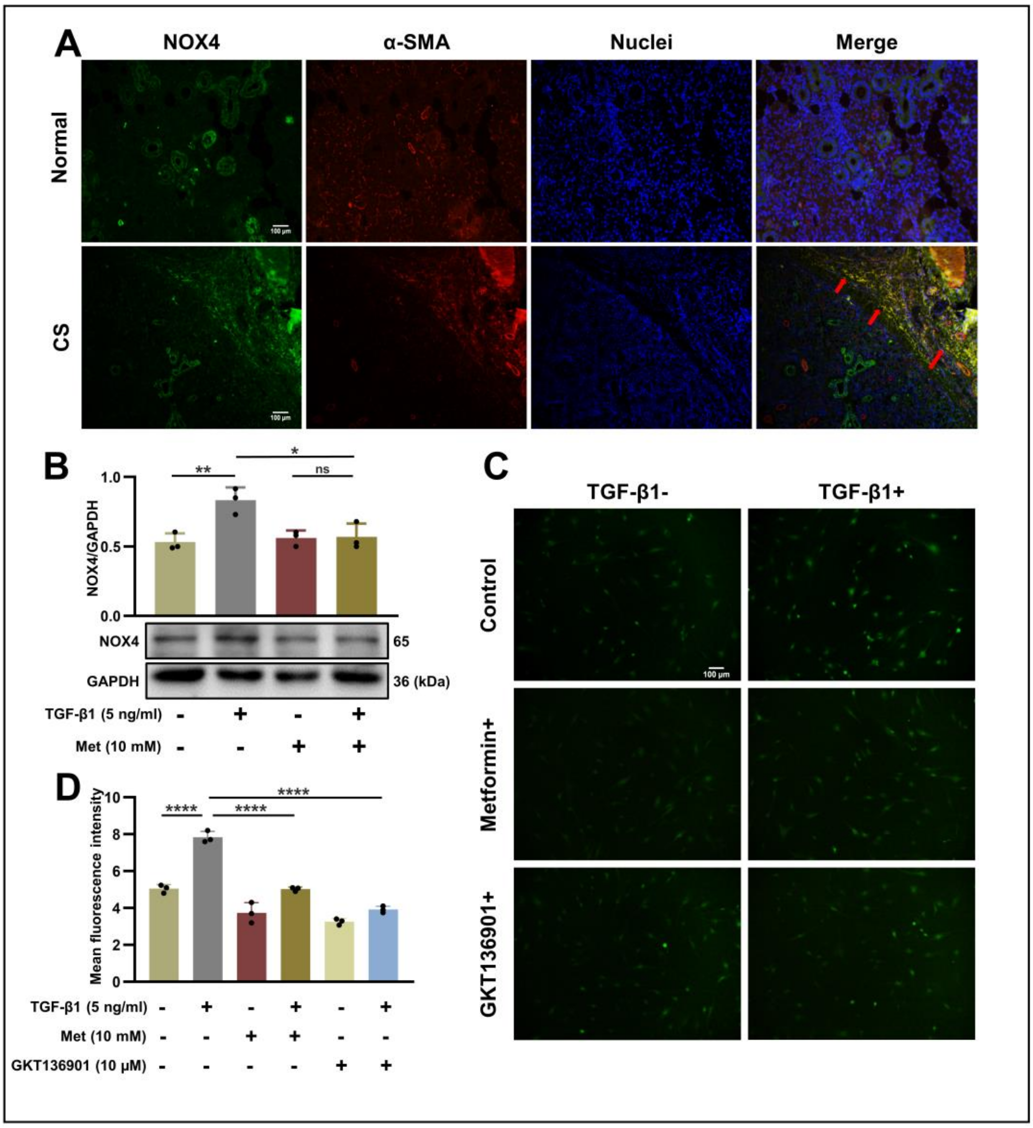
A
NOX4
α-SMA
Nuclei
Merge
Normal
CS
100 μm
B
NOX4/GAPDH
1.0
0.5
0.0
**
*
ns
NOX4
65
GAPDH
36 (kDa)
TGF-β1 (5 ng/ml)
Met (10 mM)
C
TGF-β1-
TGF-β1+
Control
Metformin+
GKT136901+
D
Mean fluorescence intensity
10
8
6
4
2
0
****
TGF-β1 (5 ng/ml)
Met (10 mM)
GKT136901 (10 μM)

Figure 3

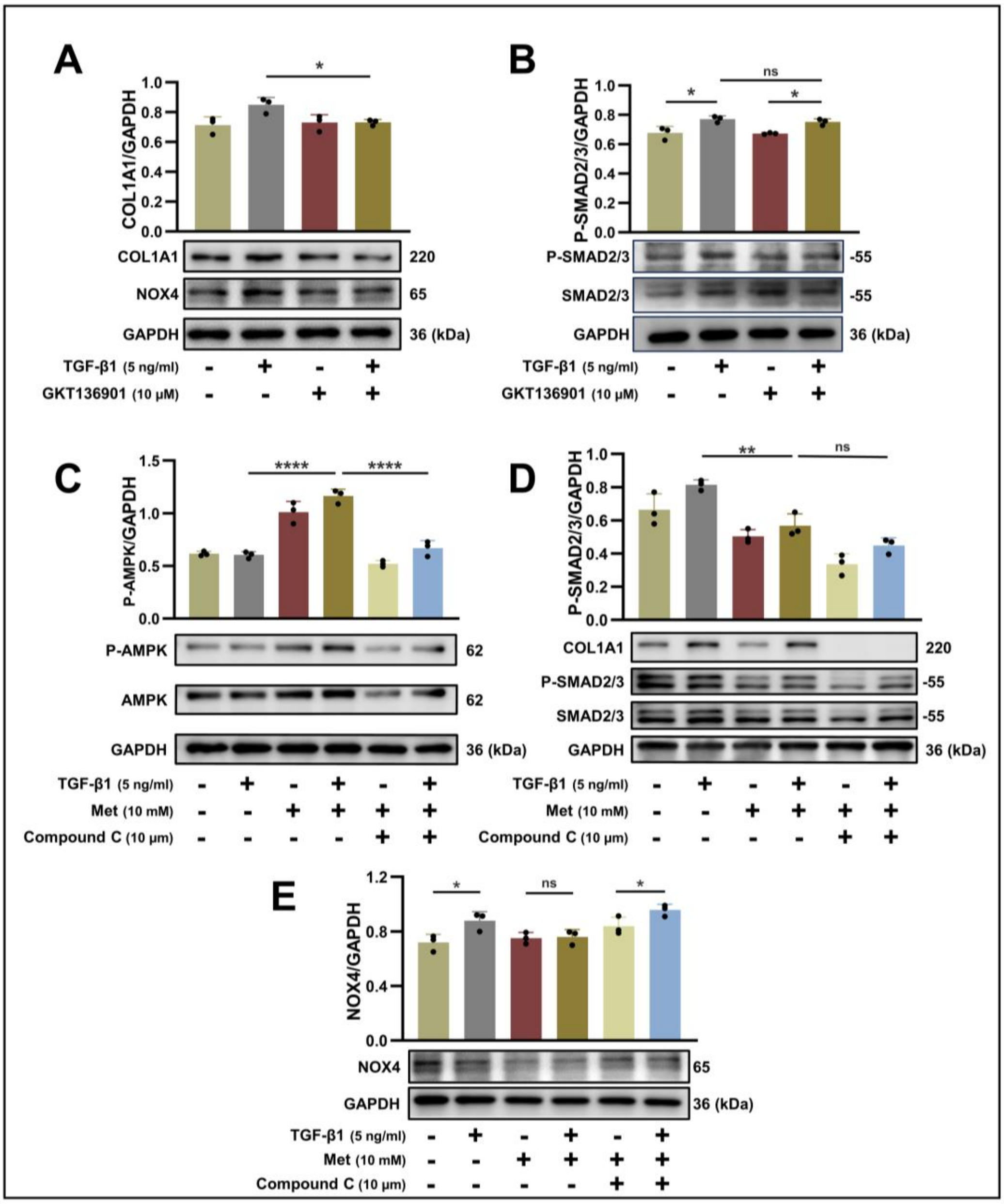

Original Figure 4

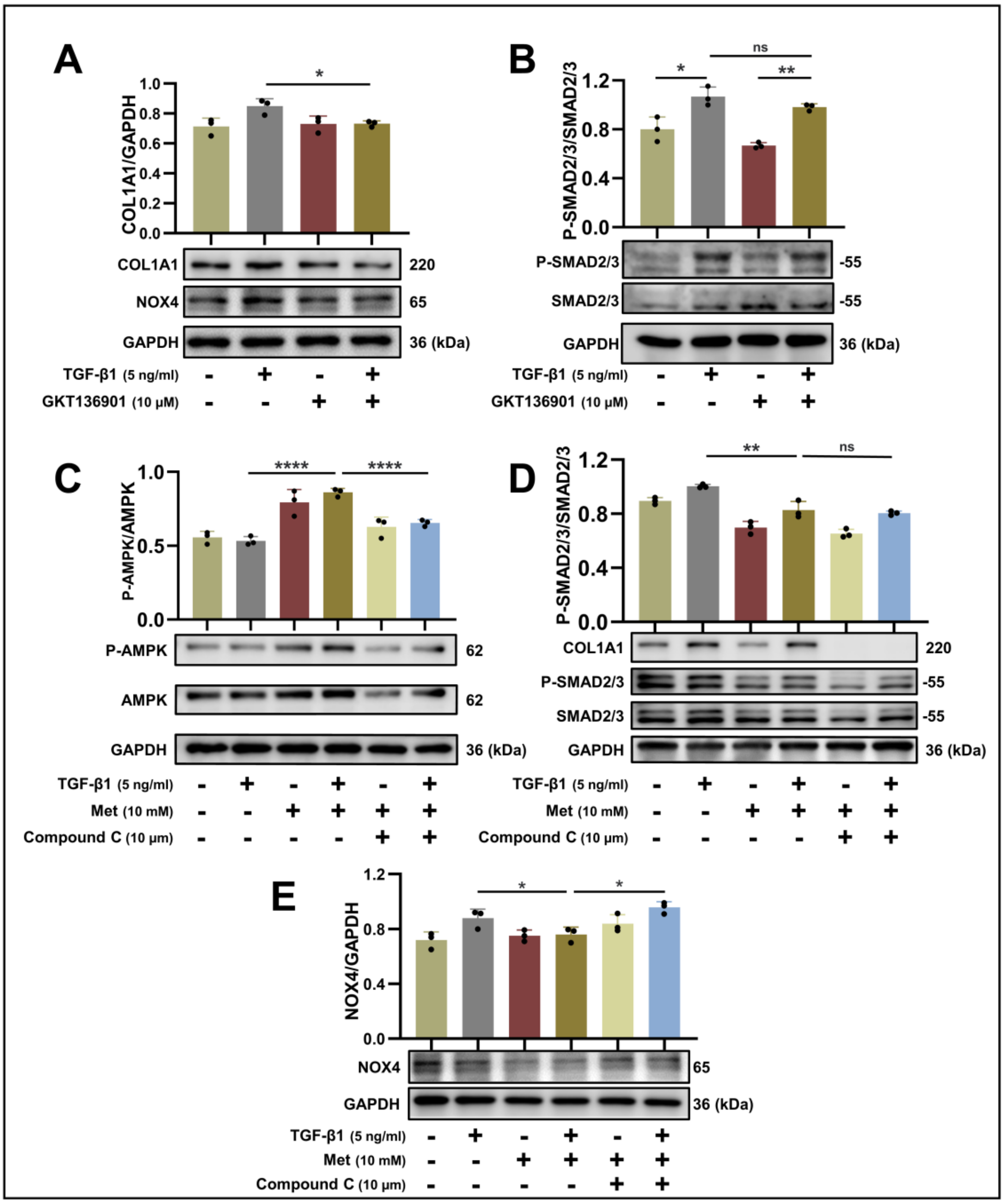

Revised Figure 4

As for Compound C's implications in **revised Figure 4C-E**, its role as a well-established AMPK inhibitor is underscored by multiple studies (PMID:36450712, PMID: 33657393, PMID: 25744403, PMID: 24357397). Our primary objective was to examine the alterations in associated indicators post-AMPK phosphorylation inhibition, and Compound C appeared fitting for this purpose. As delineated in **revised Figure 4C**, Compound C notably diminishes AMPK phosphorylation in cells, thereby obstructing its functional activities, both in terms of total and phosphorylated protein suppression.

Nonetheless, your feedback prompts us to reevaluate our experimental design to ensure a more robust and definitive interpretation of the observed effects. We are grateful for your expert guidance in this matter.

The authors should provide a quantification and statistical analysis for all immunoblots. For example, on Ln174, the authors state that Metformin "...significantly enhanced the phosphorylation of AMPK", even though there is no quantification provided for the immunoblot signal in Fig. 2D. The same applies to multiple other blots (e.g. Ln176/177, SMAD phosphorylation and protein levels in Fig. 2D).

Response: We are grateful for your keen observations regarding the quantification and statistical analysis of the immunoblots. We recognize the oversight and have now quantified the α-SMA/GAPDH and COL1A1/GAPDH ratios, and these results are presented in **revised Figures 2D**. Upon further evaluation, we discerned that while **Figure 2** displays a phenotypic indicator, AMPK serves as a mechanistic indicator. Given this differentiation and to maintain the article's logical coherence, we have opted to exclude the P-AMPK indicator in the **revised Figure 2D**.

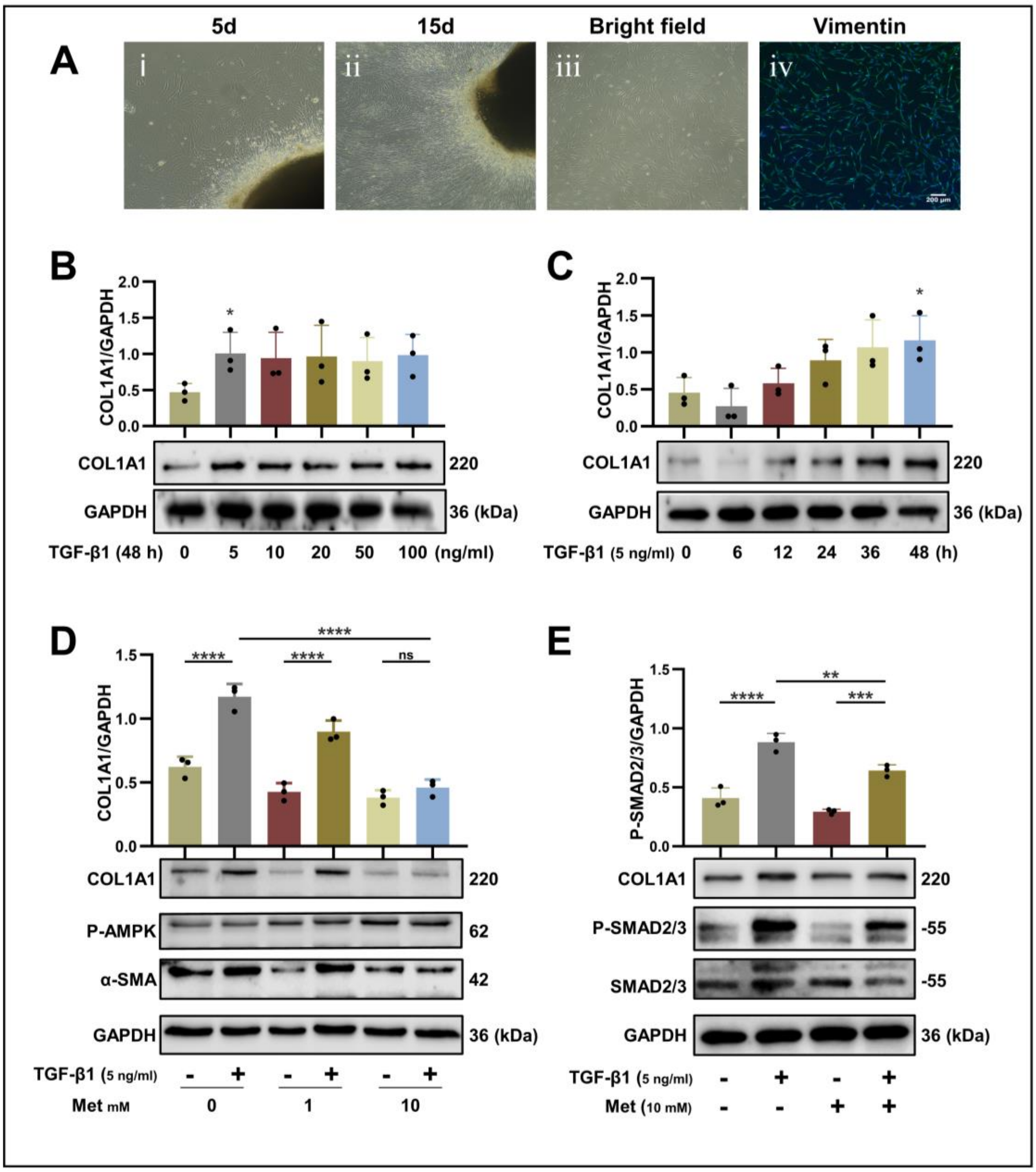

Original Figure 2

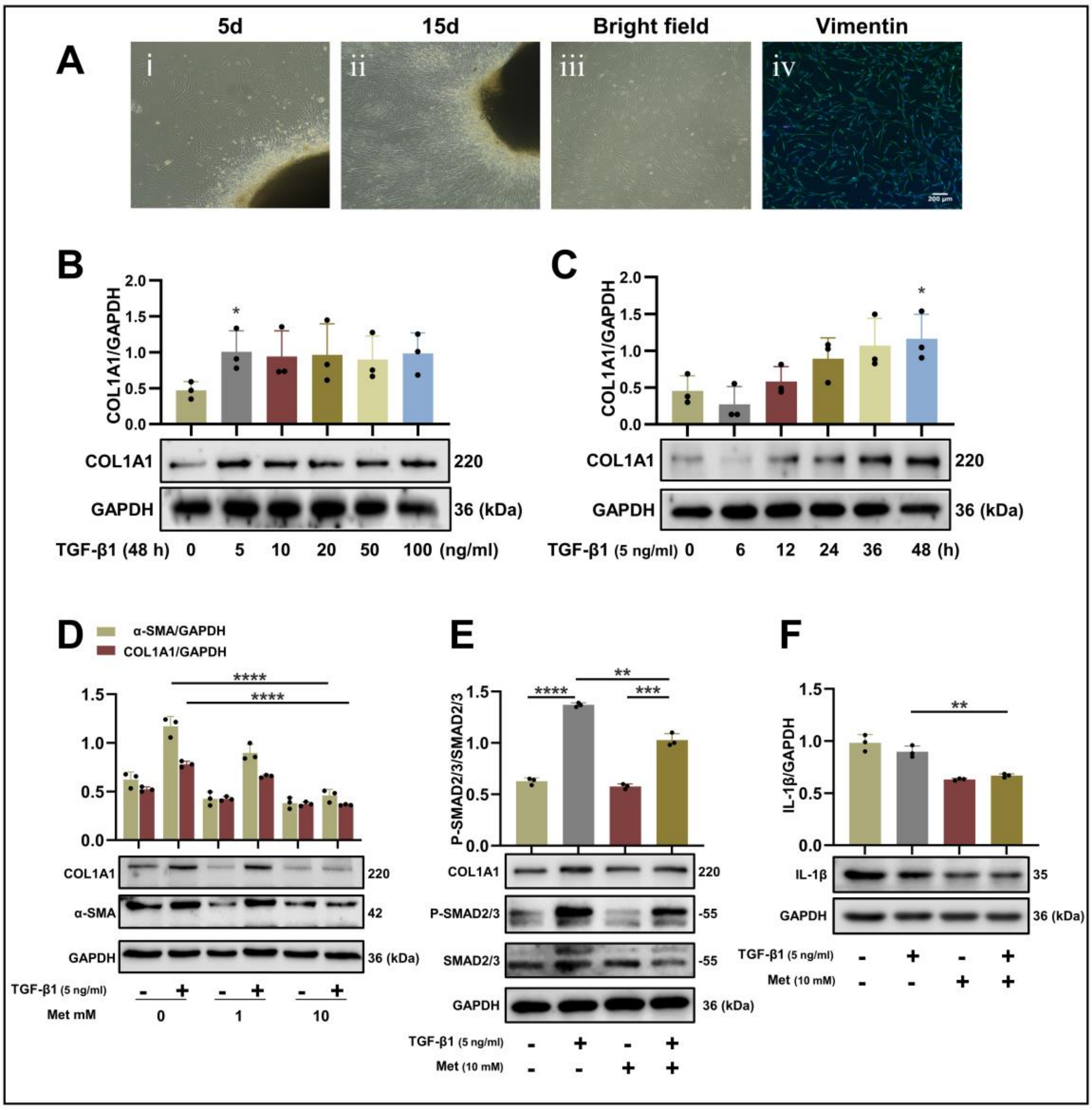

Revised Figure 2

For graphs reporting phosphorylation levels (e.g. Fig. 2E; 4B/C/D), it would be more appropriate to correct phosphorylation levels for the expression of the respective protein (e.g. P-SMAD/SMAD protein, P-AMPK/AMPK protein) rather than GAPDH, as the authors have done. For example, in Fig. 4C/5th lane, there is a significant reduction in AMPK protein levels, but not GAPDH levels.

Response: We appreciate your insightful feedback. In light of your suggestion, we have now adjusted the expression of phosphorylation levels to represent the ratio of phosphorylated protein to total protein (**revised Figure 2E, 4B-D**) rather than referencing GAPDH.

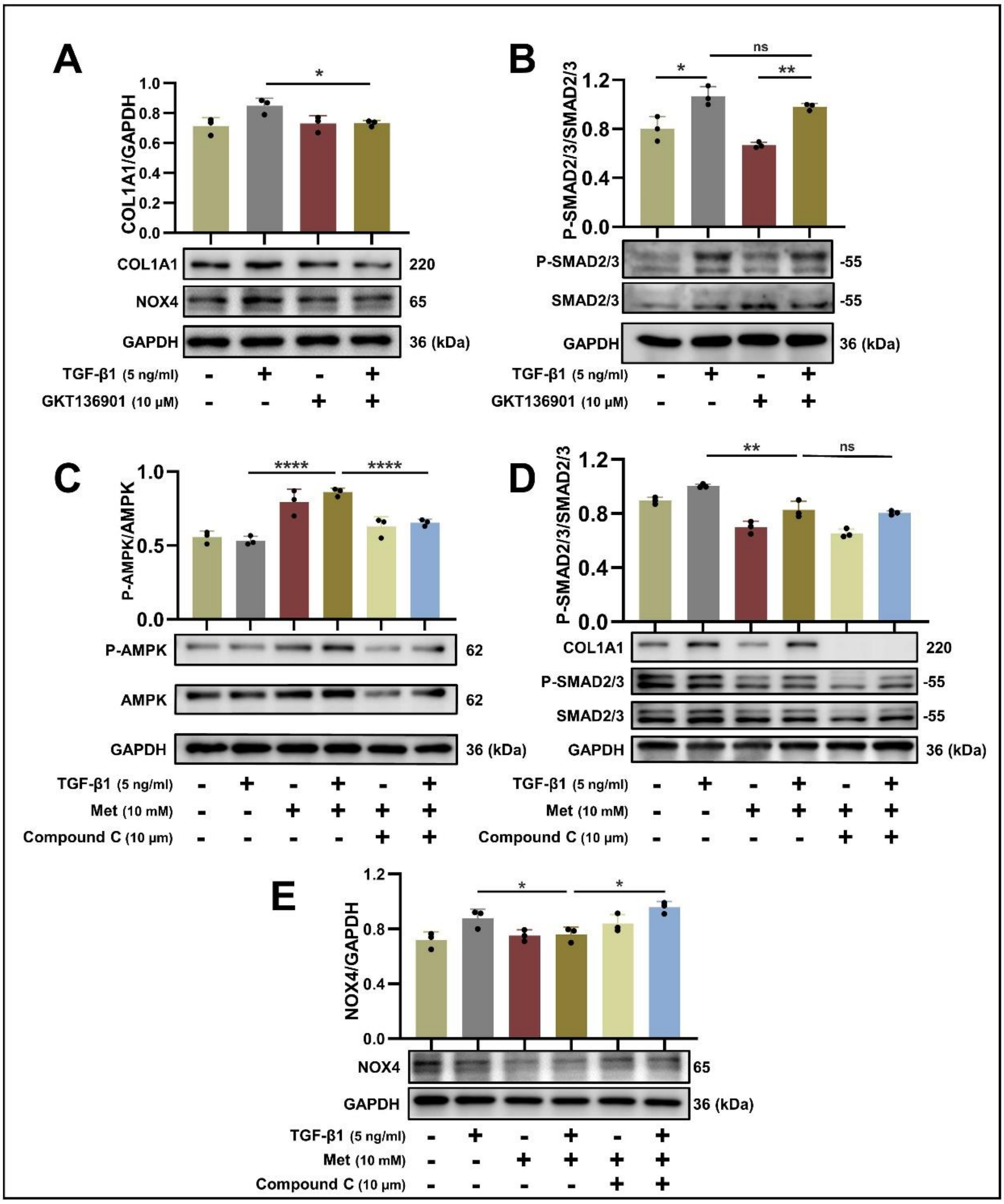

Revised Figure 4

On several occasions the immunoblots are of poor quality and/or do not reflect the changes in intensity reported in the graphs. For example, SMAD2/3 is reflected as two clear, distinct bands for some blots (e.g. Fig. 2E), but the signal is just a dirty smear of many bands in other figures (e.g. Fig. 4B). For the latter, it is unclear what the quantification really reflects. The same holds true for NOX4 (clear in Fig. 3B, smeary blob in Fig. 4E).

Response: Thank you for pointing out the inconsistencies in the clarity of the Western Blot bands across figures. We acknowledge these variations, which might have arisen from slight discrepancies in experimental conditions or batch-to-batch variations. We assure you that the statistical analysis was performed on data from three independent experiments to mitigate potential random errors. Moving forward, we will work to ensure more consistent presentation of data across figures.

On several occasions, the authors propose there are significant changes in immunoblot intensity in response to treatment, but these changes are NOT apparent even in the best (the provided) immunoblot. For example, while TGFb is shown to increase COL1A1 by about 2-fold/100% in Fig. 2A/B, the same treatment produces only a negligible (non-significant?) increase in Fig. 4A, and the authors propose significant changes in expression, while all four lanes for COL1A1 essentially look the same to me.

Response: Thank you for highlighting the discrepancies in our presentation of immunoblot intensities. The significant differences we referenced in the text pertain to statistical outcomes derived from multiple independent repetitions (3-5 times) of the experiment, rather than overt expression changes in the provided blots. We acknowledge that the difference in COL1A1 expression in **Figure 4A** (approximately 1.25-fold) may not be as evident as in **Figure 2A/B** (approximately 2-fold). Such variations could be attributed to inherent variations in experimental conditions across different batches, including cell conditions and potential changes in drug efficacy. We will strive to ensure greater consistency in our results moving forward.

Why do the original blots provided in the supplement routinely look very different in lighting/contrast/sharpness to the images provided in the manuscript? For many blots, the immunoblot signal of interest is very faint (e.g. NOX4/Fig. 4A; P-AMPK/Fig. 4C), while the loading control signal (GAPDH) is very strong for all figures. Due to saturation of the GAPDH signal upon long/strong exposure, the authors may observe small changes in the intensity of their protein of interest, while differences in loading that may be responsible are obscured by the high exposure of GAPDH.

Response: Thank you for bringing this to our attention. The discrepancies in lighting, contrast, and sharpness between the supplementary blots and those in the manuscript

arise from adjustments made to the “Original Images for Blots” file, aimed at highlighting the molecular weight markers more distinctly. We acknowledge that such adjustments might introduce visual disparities. We also understand the concerns raised regarding the saturation of the GAPDH signal. In future experiments, we will take care to ensure that our loading control signals do not overshadow the target protein signals, allowing for a more accurate representation of protein expression levels.

The authors should state whether personnel quantifying immunoblot/fluorescence signals were blinded to the treatment groups. In a similar vein, the authors should state whether personnel dissecting, staining, or quantifying salivary gland sections were blinded to the rat treatment groups (Fig. 5) or the patient group (Fig. 1).

Response: Thank you for raising this point. We can confirm that all personnel involved in quantifying immunoblot/fluorescence signals and those dissecting, staining, or quantifying salivary gland sections were blinded to the treatment groups. This information has been stated in the “**4.2. Histological staining**”, “4.3. **Immunohistochemistry (IHC) staining**”, “**4.5. Western blot analysis**” and “**4.9. Statistical analysis**” sections of our manuscript for clarity and transparency.

It is difficult to correlate the pics in Fig. 5A with the reported weights in Fig. B.

Response: Thank you for pointing this out. We recognize the discrepancies between the images in **original Figure 5A** and the data presented in **original Figure 5B**. The images in **original Figure 5A** may have been affected by different scaling ratios. Additionally, individual variations among the rats might contribute to the observed differences. To ensure clarity and consistency, we have now adjusted the images in **revised Figure 5A** to better align with the average gland weights presented in **revised Figure 5B**.

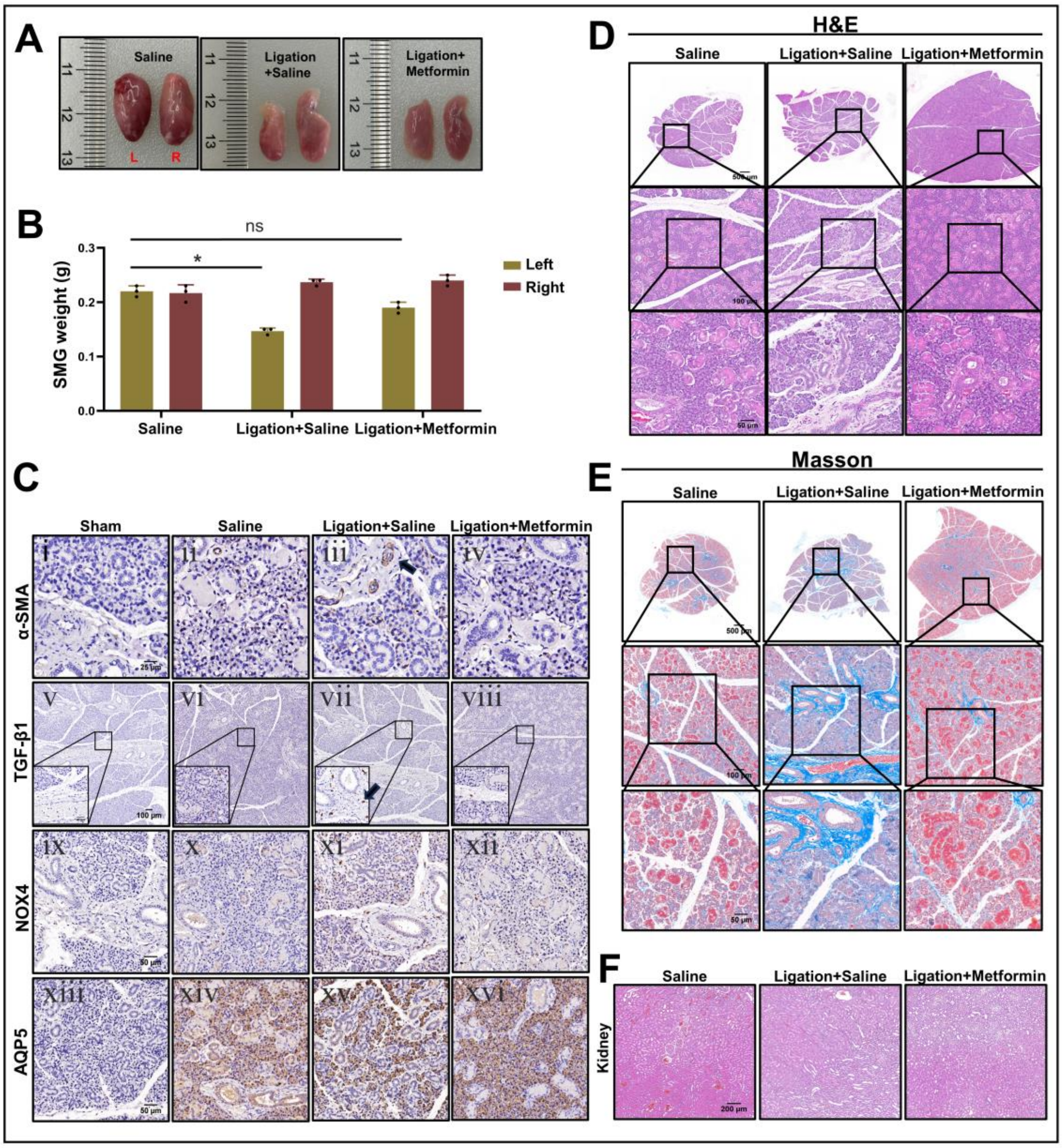

Original Figure 5

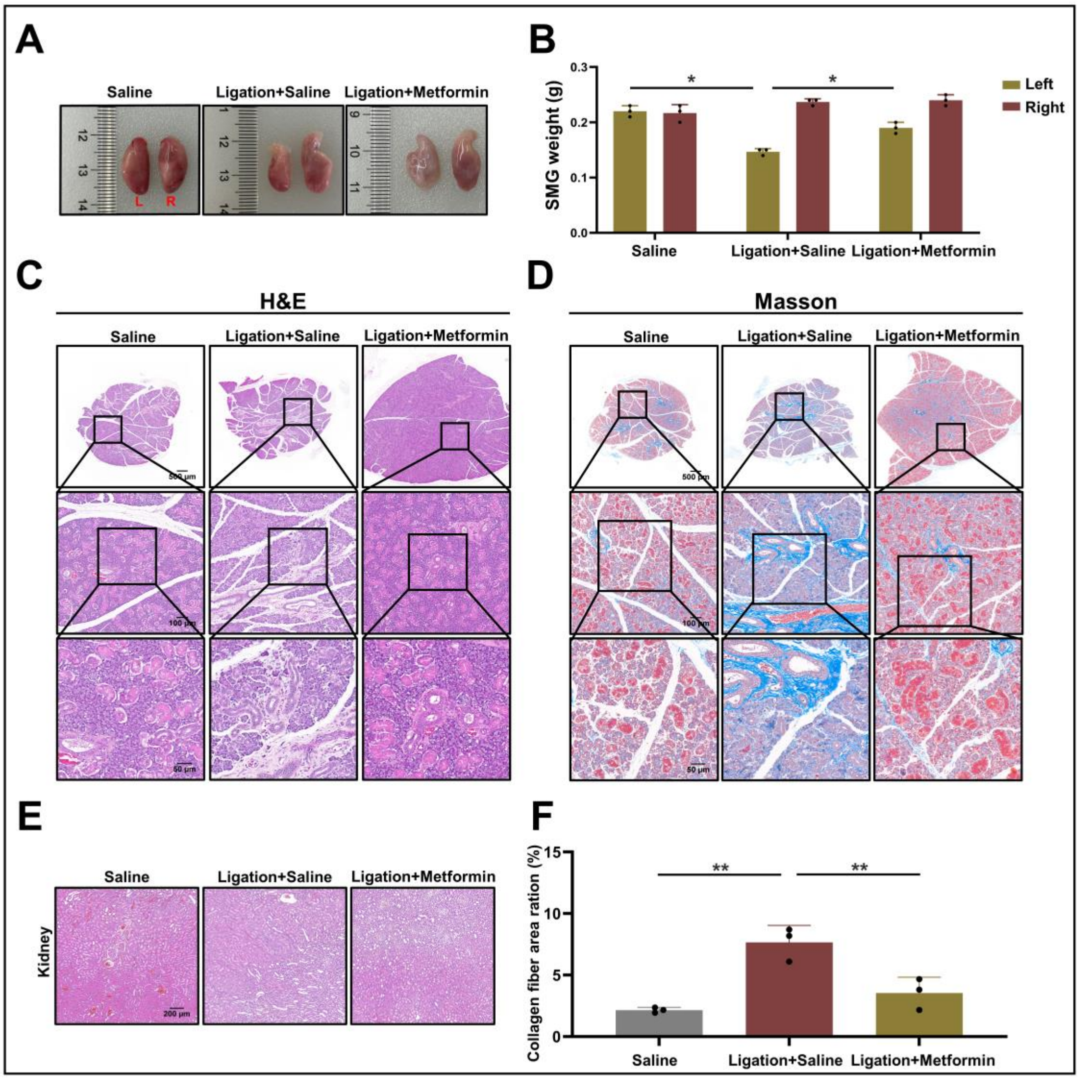

Revised Figure 5

Fig. 5D/ top right panel: why does the Lig+Met section look so different (size, density) even from Saline/no ligation?

Response: Thank you for highlighting this observation. The discrepancies in the appearance of the Lig+Met section in **original Figure 5D** compared to the Saline/No ligation may stem from variations in the tissue sectioning process, where the cross-section might not have been taken at its maximum size. Additionally, inherent individual differences among the rats could also play a role in the observed differences. We will ensure more consistent sectioning in future experiments.

Fig. 5C-F, please provide quantifications and statistical analyses.

Response: Thank you for pointing this out. We have now provided quantitative statistical analyses for **original Figure 5C/E**.

Specifically, the **revised Figure 6** presents the quantification for the **original Figure 5C**, the **revised Figure 5D** offers the quantification for the **original Figure 5E**, and we have included **revised Figure 5F** to provide a quantitative analysis corresponding to the **original Figure 5E**.

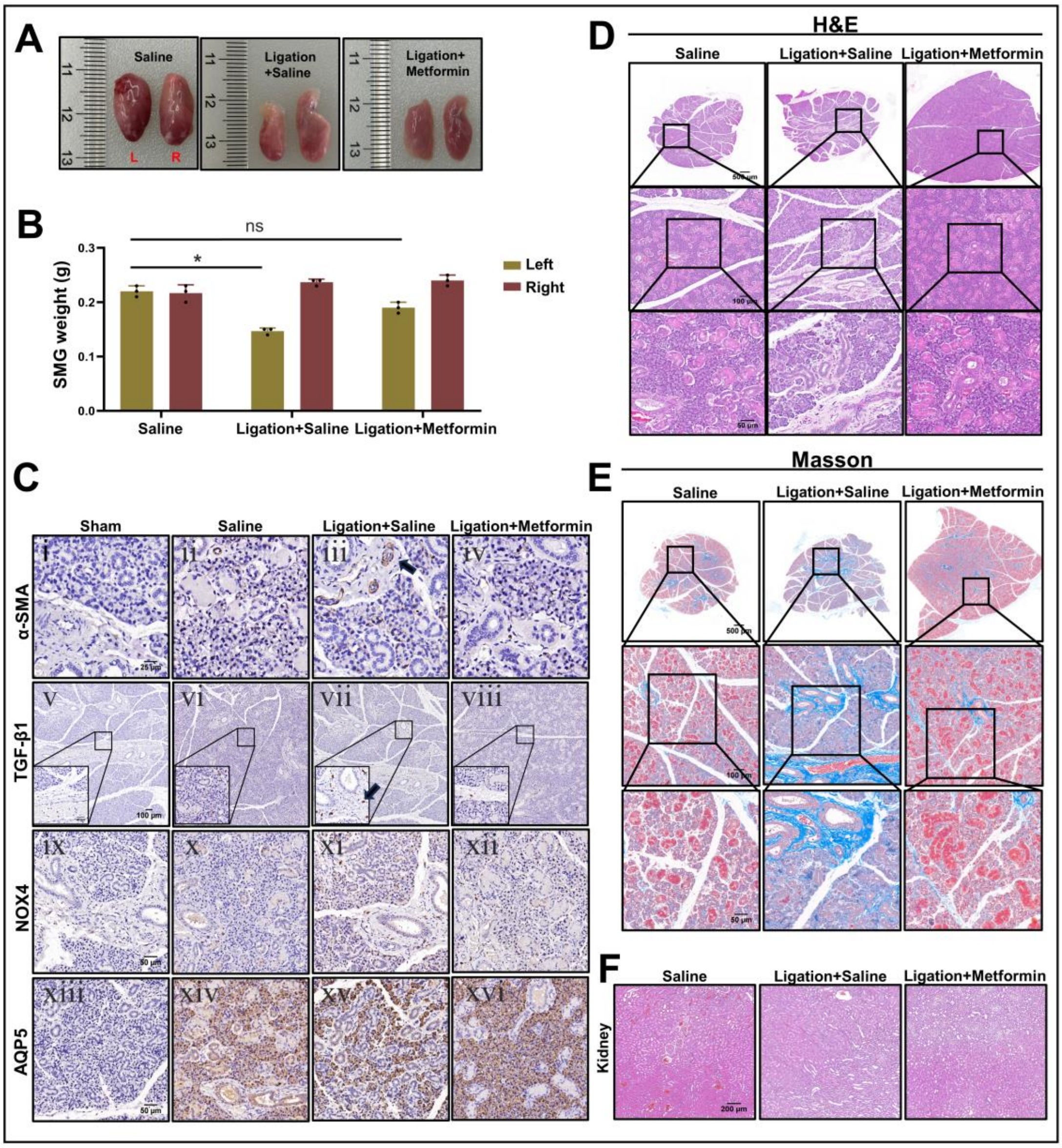

Original Figure 5

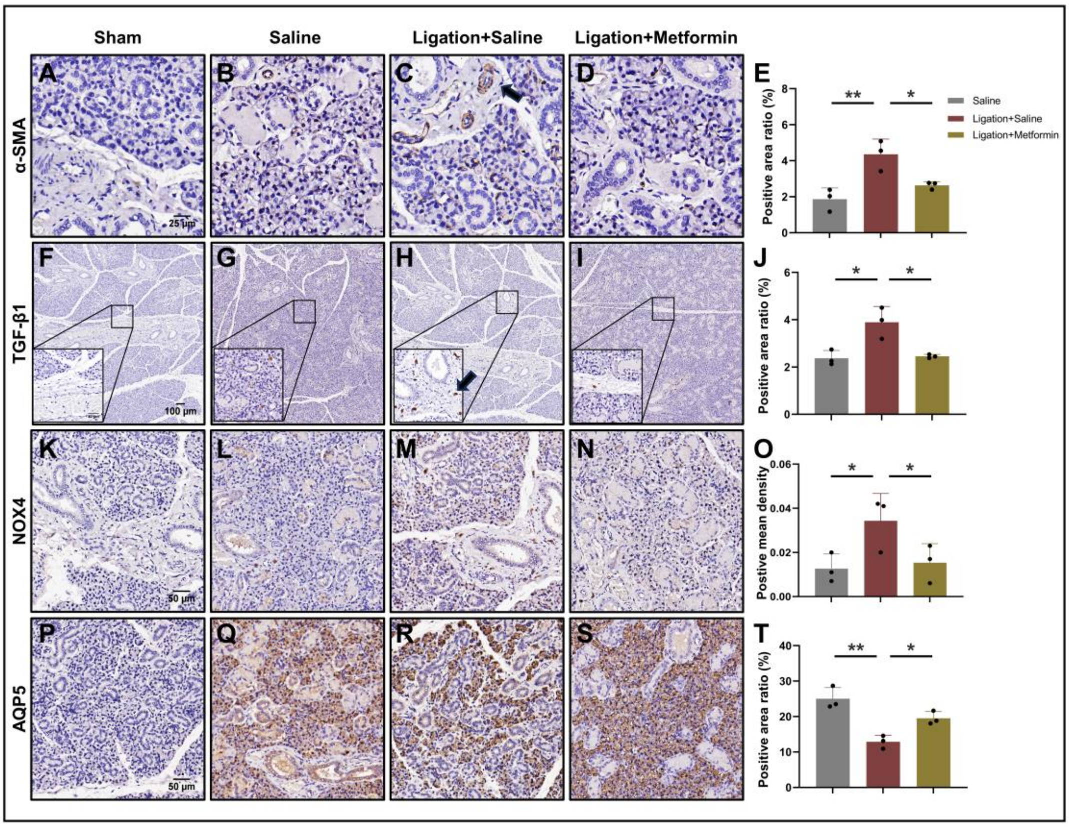

Revised Figure 6

Discussion: Metformin may act on a number of cells (e.g. immune or vascular cells; e.g. PMID: 31164166) to produce potentially therapeutic benefits in humans and animals. The authors may consider acknowledging that the benefits observed in the rat model may result, in small or large parts, through the action of Metformin on cells other than fibroblasts

Response: Thank you for pointing out the potential broader actions of Metformin. We appreciate your reference to the relevant literature. In light of your suggestion, we have expanded our discussion in the third to last paragraphs of the **Discussion** section to acknowledge that the therapeutic benefits of Metformin observed in our rat model might be mediated not only by its effects on fibroblasts but also potentially on other cell types such as immune or vascular cells.

Ln 368/"restoration" The only conclusion from the current study is that Metformin may "prevent" some damage/fibrosis induced by TGFb/duct ligation. Whether Metformin may "reverse" pre-existing injury or "re-store" normal function, is a different question

that remains to be tested

Response: Thank you for your insightful observation regarding the use of the term "restoration". We concur with your assessment and recognize the distinction between "preventing" damage and "reversing" pre-existing injury. We have revised the wording in the penultimate paragraph of the **Discussion** section to more accurately reflect our findings and have avoided the use of the term "restore" in this context. We appreciate your guidance in enhancing the clarity of our manuscript.

We deeply value your expert guidance which has been instrumental in refining our work. Your time and expertise are genuinely appreciated. Should there be further areas of improvement in our manuscript, we warmly welcome your suggestions.

Response to Reviewer 3's comments:

Dear Reviewer,

Thank you for your insightful comments and valuable feedback. We genuinely appreciate the time and effort you dedicated to reviewing our work.

Major comments:

In this work, it was suggested that the effect of metformin on preventing fibroblast to myofibroblast differentiation depends on Nox4 and ROS production. However, the authors did not provide evidence of a direct connection. To determine the effect of metformin in the absence of NOX4, further evaluation is necessary.

Response: Thank you for emphasizing the importance of establishing a direct connection between metformin's effect and the role of NOX4 and ROS production.

Our study primarily investigates the anti-fibrotic properties of metformin on the SMAD2/3 pathway. To further elucidate the underlying mechanisms, we assessed the ancillary role of NOX4. By treating cells with a NOX1/4 inhibitor, GKT136901, we noted a reduction in both ROS levels and COL1A1 protein concentrations, mirroring metformin's therapeutic impact. Based on these observations, we postulate a potential relationship between metformin's effect on the TGF-β fibrosis response and NOX4.

However, we acknowledge the absence of conclusive evidence for a direct link between metformin and NOX4. We have highlighted this limitation in our discussion and recognize the need for further experiments to conclusively elucidate this relationship.

As ROS production results from dysfunctional mitochondria, it is important to assess the mitochondria's function in terms of mass, shape, and metabolism. If ROS is a primary trigger for inducing fibroblast to myofibroblast differentiation, it is important to investigate if the phenotype changes in the presence of NAC rescue.

Response: Thank you for emphasizing the significance of assessing mitochondrial function in relation to ROS production and its potential role in fibroblast to myofibroblast differentiation. We recognize the depth and relevance of your insights.

In our current study, while we have observed the potential involvement of NOX4 in the process, our primary focus was on the effect of metformin on fibrosis. To reflect the exploratory nature of our findings, we have appended "**A preliminary study**" to the **Title**. We genuinely appreciate your suggestion on evaluating the phenotype changes in the presence of NAC. We believe it would be a valuable avenue for our future in-depth research, allowing us to further elucidate the role of ROS in fibrosis.

Additionally, if metformin controls Nox4 through AMPK, does the effect of metformin weaken in the absence of AMPK? It is noteworthy that AMPK phosphorylation levels are high and similar to baseline.

Response: Thank you for drawing attention to the potential relationship between metformin's effect and AMPK presence. We have indeed conducted preliminary experiments using the AMPK inhibitor (Compound C), which suggests a possible connection between metformin's action and AMPK. However, we have not performed exhaustive tests to definitively validate this relationship. Recognizing the significance of this point, we've added a discussion on this limitation in the penultimate paragraph of the **Discussion** section. We appreciate your valuable feedback in enhancing the comprehensiveness of our manuscript.

In vivo studies have demonstrated that metformin treatment reduces the fibrotic response in a ligation model. However, the authors have not shown a direct involvement of AMPK-NOX4 regulation. This issue could be resolved by using a conditional KO model.

Response: Thank you for highlighting the need for a more direct demonstration of AMPK-NOX4 regulation. While our study did show the potential therapeutic effects of metformin on salivary gland fibrosis, we concur that a direct link between AMPK-NOX4 and the observed outcomes was not thoroughly established. Using a conditional KO model, as you've suggested, could indeed provide more definitive insights. We

have acknowledged this limitation in the penultimate paragraph of the **Discussion** section. Moving forward, we are considering the use of KO animal models to delve deeper into the mechanisms through which metformin acts on salivary gland fibrosis. Your feedback is invaluable in guiding our future research endeavors.

Minor comment:

Fig 1. Consider changing change the term "expression of fibrosis" in Fig 1 to "evaluation of fibrotic response".

Response: Thank you for your valuable suggestion. We have revised the original text to read "**evaluation of fibrotic response**" instead of "**expression of fibrosis**".

Additionally, in Fig 2D, the authors may have overlooked adding the blot of the corresponding Total AMPK.

Response: Thank you for pointing out this. Upon review, we recognize that while **original Figure 2** primarily displays phenotypic indicators, AMPK serves as a mechanistic indicator. To maintain consistency and logical coherence within the article, we have decided to omit the P-AMPK indicator. We appreciate your diligence in drawing our attention to this matter.

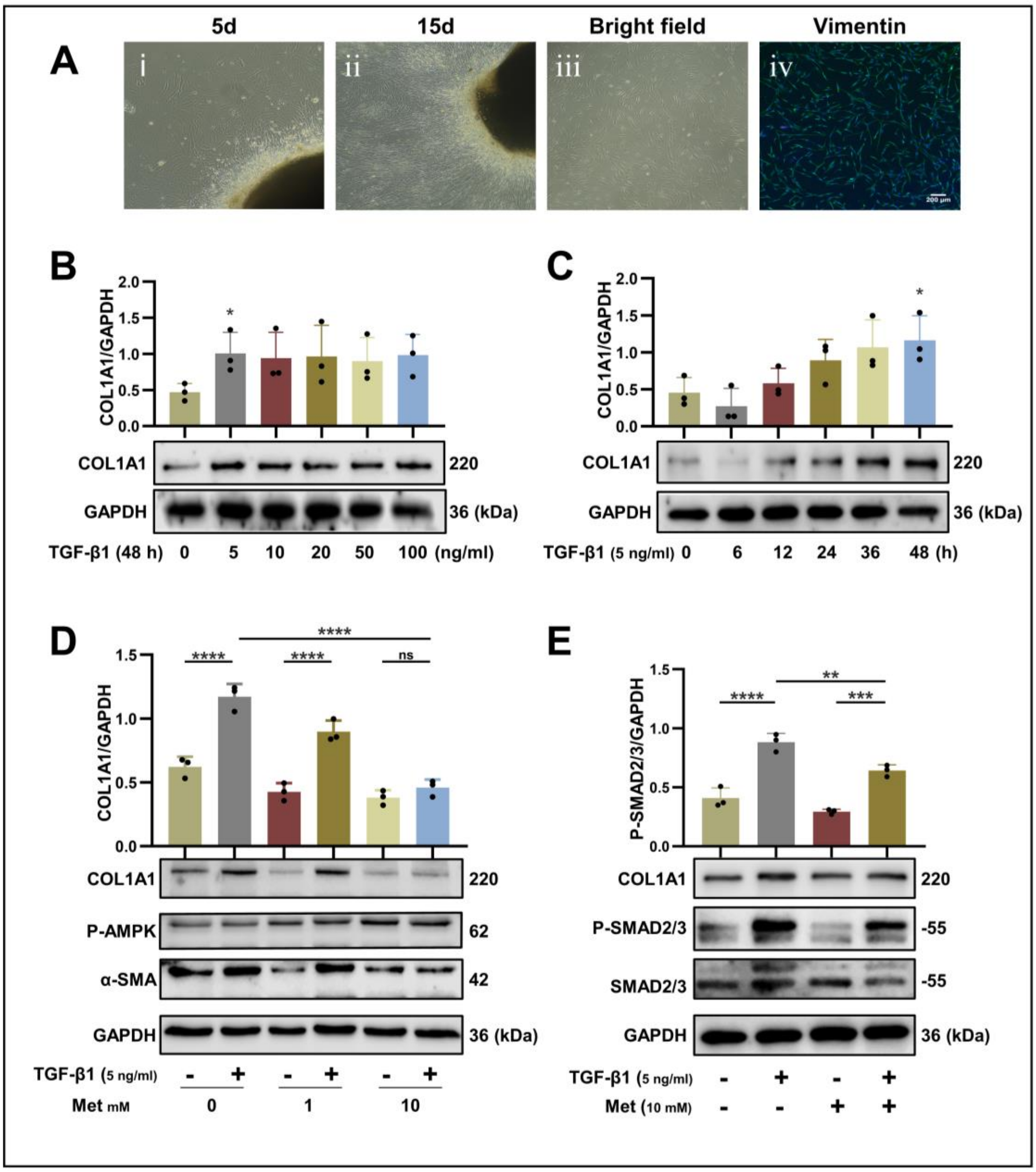

Original Figure 2

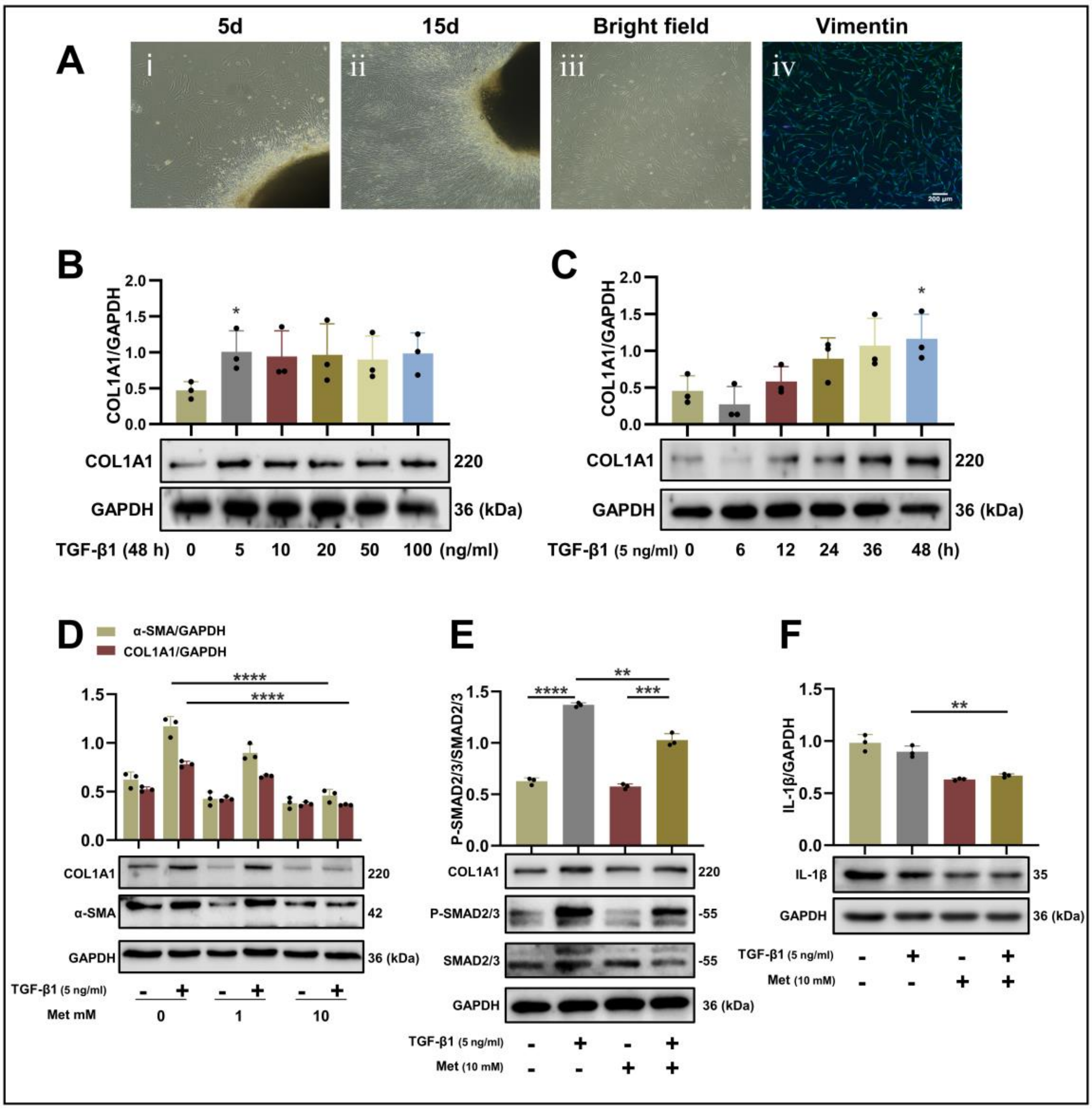

Revised Figure 2

We deeply value your expert guidance which has been instrumental in refining our work. Your time and expertise are genuinely appreciated. Should there be further areas of improvement in our manuscript, we warmly welcome your suggestions.

Round 2：

Response to Reviewer 2's comments:

The authors have made appropriate changes to the manuscript in response to a few comments, while the author's reply to most comments was to consider them for future experiments, in which they promise to explore the mechanism of Metformin's action in salivary gland dysfunction in more detail. In line with this idea, the authors have revised the title of their current manuscript as "a preliminary study."

While "preliminary studies" have their place in the literature, I find this approach risky in the case of this particular manuscript, because the authors propose a molecular mechanism for how metformin may alleviate salivary gland fibrosis, and "mechanisms" can be either correct or incorrect, but should not be "preliminarily correct".

I am thus not enthusiastic about the publication of the manuscript in its current form. One approach to resolve this could be to simply publish the original Reviewers' comments with the Authors' responses as a Supplement to the manuscript. While unusual, this would serve the future readers of the manuscript by highlighting some of the manuscript's remaining limitations and weaknesses, while not requiring further experiments or revisions to the manuscript itself.

Response: Dear Reviewer,

Thank you for your critical evaluation and the thoughtful suggestion provided. We understand your concerns regarding the designation of our study as "preliminary" and the implications it may have on the proposed molecular mechanisms.

After careful consideration, we agree that publishing the original reviewers' comments alongside the authors' responses could indeed offer transparency and serve as a beneficial resource for future readers by openly addressing the current limitations and areas for further research. This action will allow us to contribute to the scientific discussion while acknowledging the need for more detailed future investigations.

We are grateful for the opportunity to improve our work with your guidance.

Alternatively, I would like to ask the authors for more revisions addressing my prior comments more comprehensively, including:

1. The metformin dosage disparity between the in vivo and in vitro experiments, which makes it likely that metformin may act in vivo via molecular mechanisms that are distinct from those explored in cell culture. This may simply be accomplished by adding some related text to the Discussion section of the manuscript;

Response: We are grateful for your astute observation regarding the dosage discrepancy between in vivo and in vitro experiments of metformin and its potential implication on the mode of action. In accordance with your suggestion, we have expanded the **Discussion** section to address this issue more comprehensively.

In the revised manuscript, the seventh paragraph of the **Discussion** now includes an analysis of the disparity in metformin dosages used in our experiments. We acknowledge that this variance might suggest the activation of different molecular pathways *in vivo* compared to those identified in cell culture studies. We also clarify that identifying the exact concentration of metformin that elicits anti-fibrotic effects in the rat submandibular gland via oral administration remains challenging. Furthermore, we have highlighted the necessity for additional research to ascertain whether the mechanisms observed in vitro correlate directly with the *in vivo* actions.

"*It is important to note that the precise concentration of metformin required for anti-fibrotic effects in the rat submandibular gland, when administered orally, remains elusive. Additionally, whether the mechanism of action at this concentration is fully consistent with in vitro observations warrants further investigation.*"

2. Perform some of the experiments proposed in the original review to validate the proposed mechanism of Metformin's action, including more extensive use of concentration-response curves to ensure that all of metformin's effects occur via the

same effector and hence at the same dose; show that metformin has no additional effects in the presence of GKT136901; and/or show the effect of Compound C by itself alongside its effects in the presence of metformin.

Response: To further elucidate the mechanism of metformin's action, we investigated its inhibitory effect on fibrosis across a broader range of concentrations and present the results in the updated **Supplemental Figure 1A**.

Additionally, in the presence of GKT136901, we also sought to determine whether metformin may elicit additional anti-fibrotic effects to fully exhibit its mechanism of action. The results are shown in **Supplemental Figure 1B-D**, and discussed in **Section 2.6** of this article.

"*To further explore the role of NOX4 in the anti-fibrotic process of metformin, we also examined whether metformin could further enhance the anti-fibrotic effect in the presence of GKT136901. The results showed that in addition to GKT136901 inhibiting NOX4 expression and thereby producing an anti-fibrotic effect, metformin further inhibited fibrosis (Supplementary Figure 1B-D). This suggests that inhibition of NOX4 expression may only be one of the pathways through which metformin inhibits fibrosis, and metformin also exerts anti-fibrotic effects through other pathways.*"

These findings further suggest that NOX4 may be involved in metformin's anti-fibrotic effect, and that metformin may also act through other pathways beyond this one.

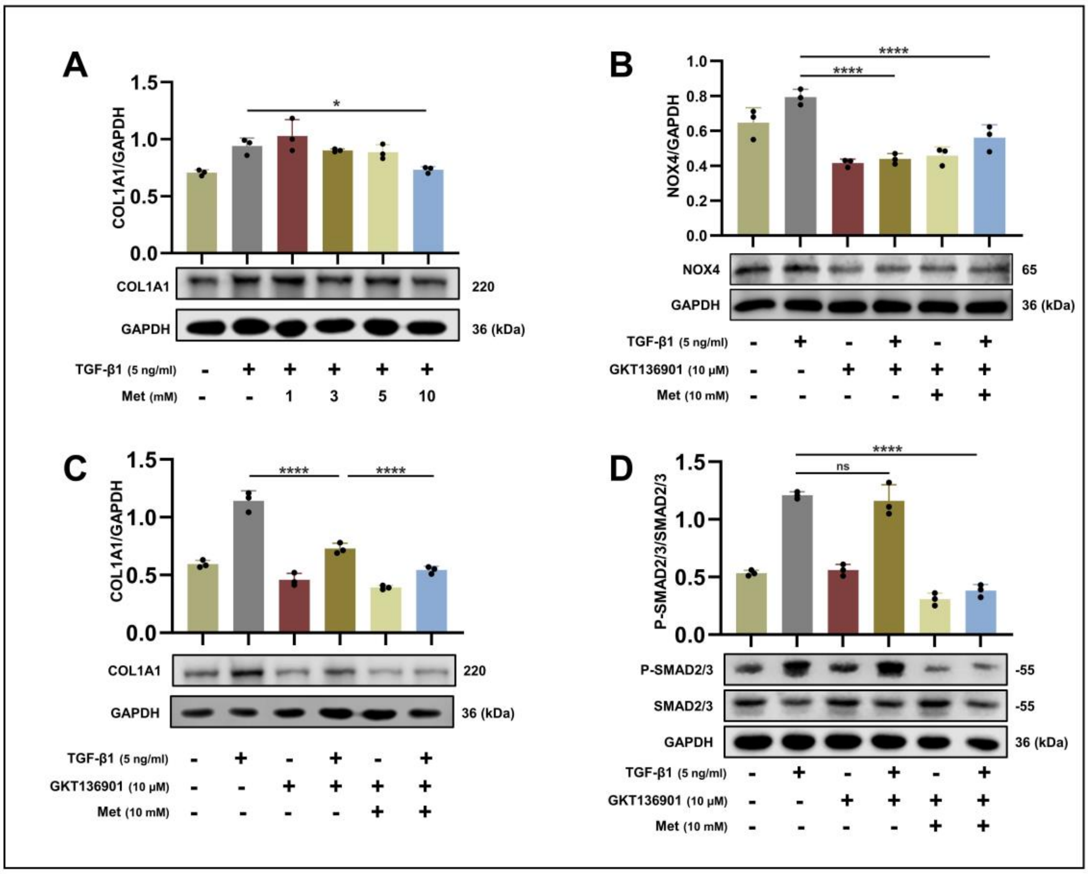

Supplemental Figure 1 (in updated version)

3. If the authors have data showing that a 24h metformin pre-treatment is not required for its efficacy (as they state in the Response to review), please include these data in the manuscript (perhaps as a Supplement).

Response: We appreciate your insightful inquiry regarding the necessity of a 24h metformin pre-treatment in our study. In the initial stages of our experimental design, we drew upon existing literature that predominantly utilized a pre-treatment approach with metformin, which informed our decision to adopt this methodology.

Upon reflection, we realize that our previous response may have conveyed a lack of available data on this aspect of our study. We would like to clarify that during our investigations, incidental observations suggested that metformin's efficacy was not solely dependent on a 24-hour pre-treatment regimen. However, these observations were not systematically recorded as they were beyond the initial scope of our experimental design.

In the manuscript, we have clarified in the second-to-last paragraph of the **Discussion** that our reference to similar outcomes without pre-treatment was anecdotal and not derived from structured experimentation.

"*It is worth noting that in our cell experiment, metformin was pre-treated for 24 hours, which may have caused a subtle change in the cellular state compared to direct treatment. This alteration could also play a role in the anti-fibrotic action of metformin.*"

We apologize for any confusion our previous response may have caused and thank you for the opportunity to rectify this matter.

4. Please address some of the issues with the quality of immunoblot bands (P-SMAD2/3 and Nox4), if necessary with new/additional experimentation. If a blot shows a smear, rather than a clear individual band, it is not clear to me how the authors distinguished the true immunoblot signal from the potentially non-specific background for their quantification.

Response: Thank you for pointing out the issues with the clarity of our immunoblot bands for P-SMAD2/3 and Nox4. We understand the importance of discernible bands for the accurate interpretation of immunoblot data.

In response to your comment, we have conducted additional experiments to improve the quality of these blots. The revised immunoblots now exhibit more defined bands, allowing for more precise quantification. These updated results have been incorporated into the manuscript in **Figure 4B and 4E**.

We appreciate your vigilance in ensuring the quality of our data presentation and hope that the revised figure meets the high standards required for publication.

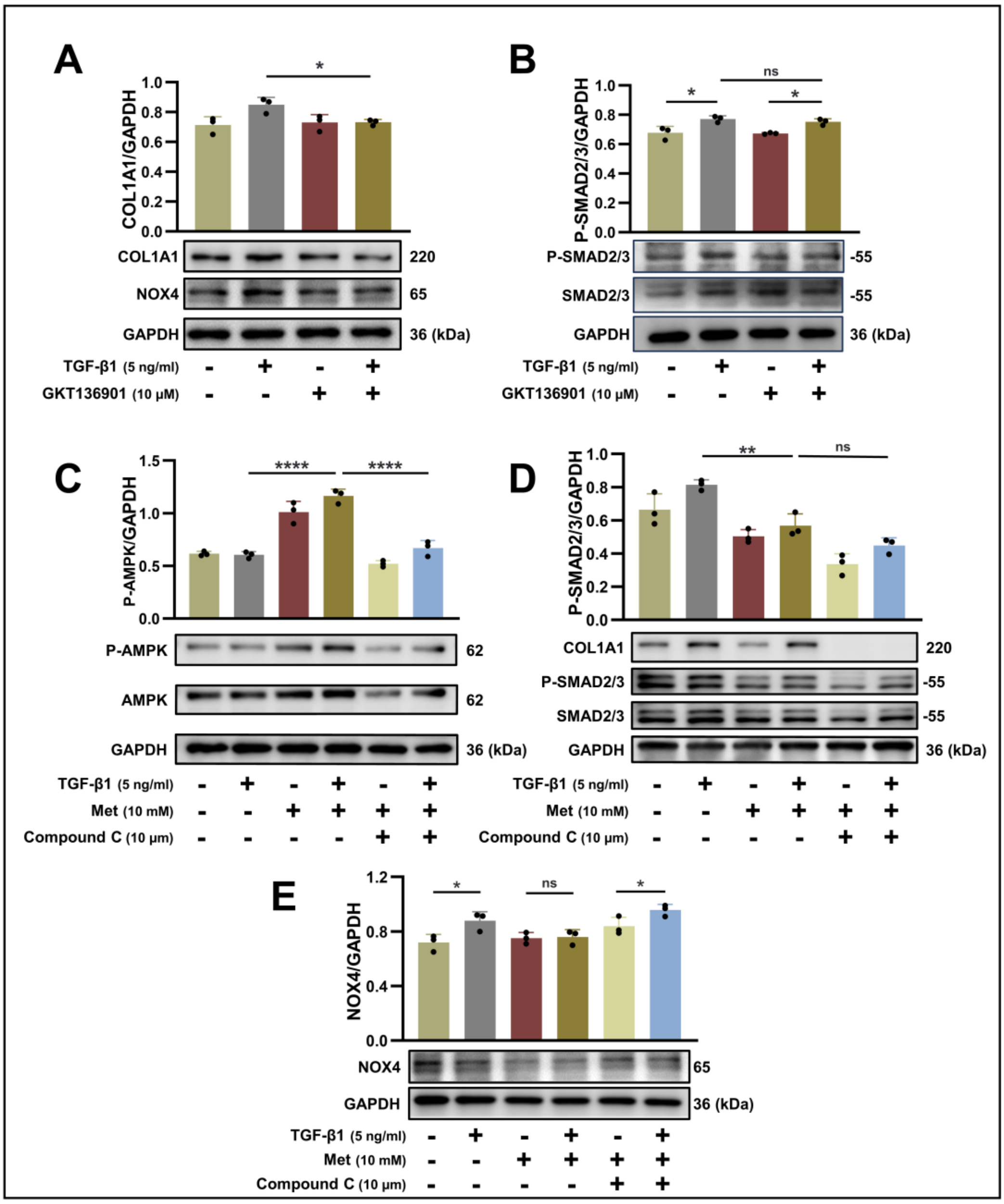

Original Figure 4

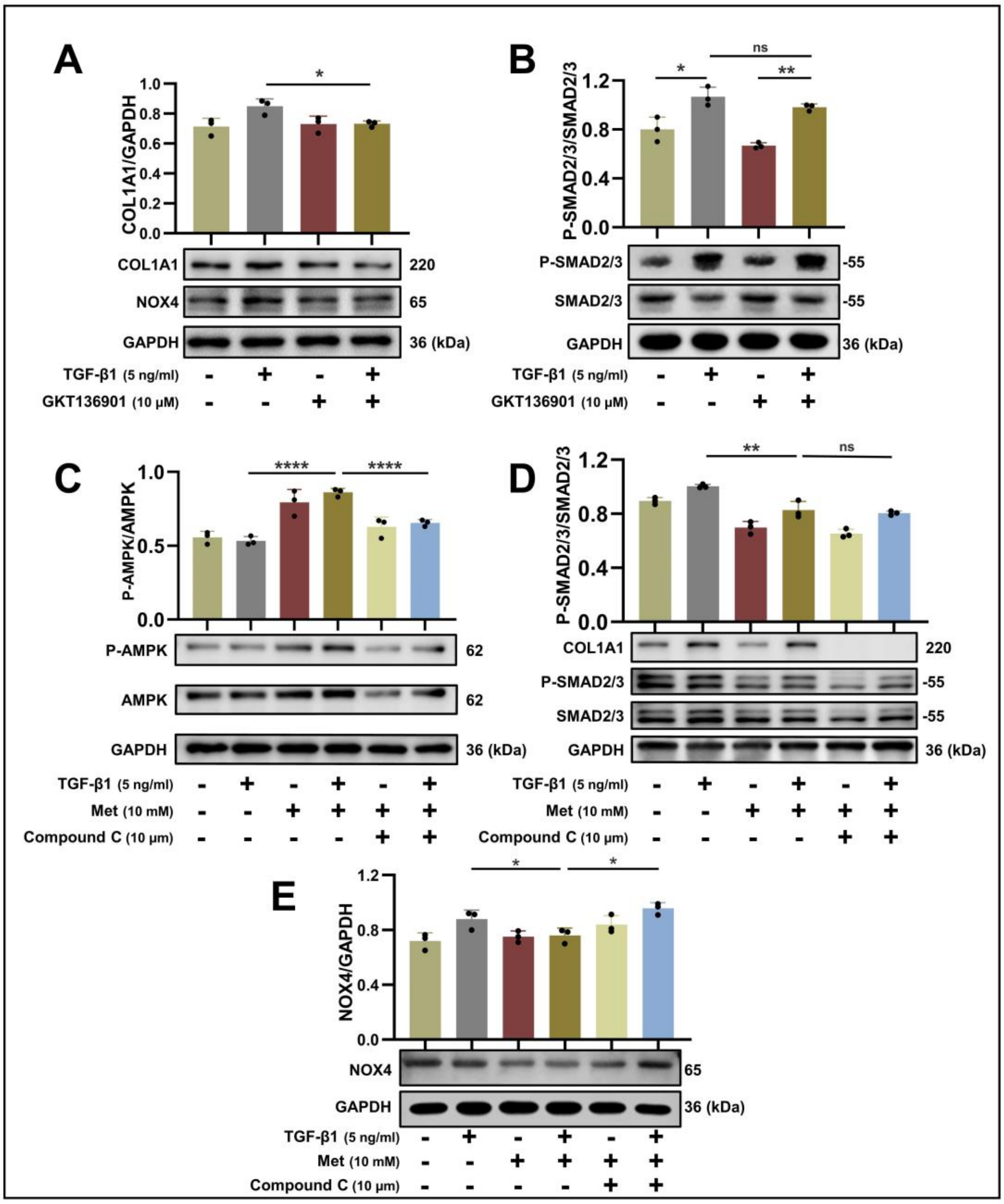

Revised Figure 4

We deeply value your expert guidance which has been instrumental in refining our work. Your time and expertise are genuinely appreciated. Should there be further areas of improvement in our manuscript, we warmly welcome your suggestions.

Response to Reviewer 3's comments:

Minor comments:

Add scale bars to every figure and use a larger font size.

Response: Dear Reviewer,

We are grateful for your constructive feedback. In response to your comments, we have now incorporated scale bars into all figures to aid in data interpretation. Additionally, the font size has been increased for better readability.

Your attention to detail has significantly contributed to the overall quality of our manuscript.
